# Maximizing Nanoscale Downshifting Energy Transfer in a Metallosupramolecular Cr(III)–Er(III) Assembly

**DOI:** 10.1021/acs.inorgchem.4c02397

**Published:** 2024-08-20

**Authors:** Maxime Poncet, Céline Besnard, Juan-Ramón Jiménez, Claude Piguet

**Affiliations:** † Department of Inorganic and Analytical Chemistry, University of Geneva, 30 quai E. Ansermet, CH-1211 Geneva 4, Switzerland; ‡ Laboratory of Crystallography, University of Geneva, 24 quai E. Ansermet, CH-1211 Geneva 4, Switzerland; § Departamento de Química Inorgánica, Facultad de Ciencias, Universidad de Granada and Unidad de Excelencia en Química (UEQ), Avda. Fuente Nueva s/n, 18071 Granada, Spain

## Abstract

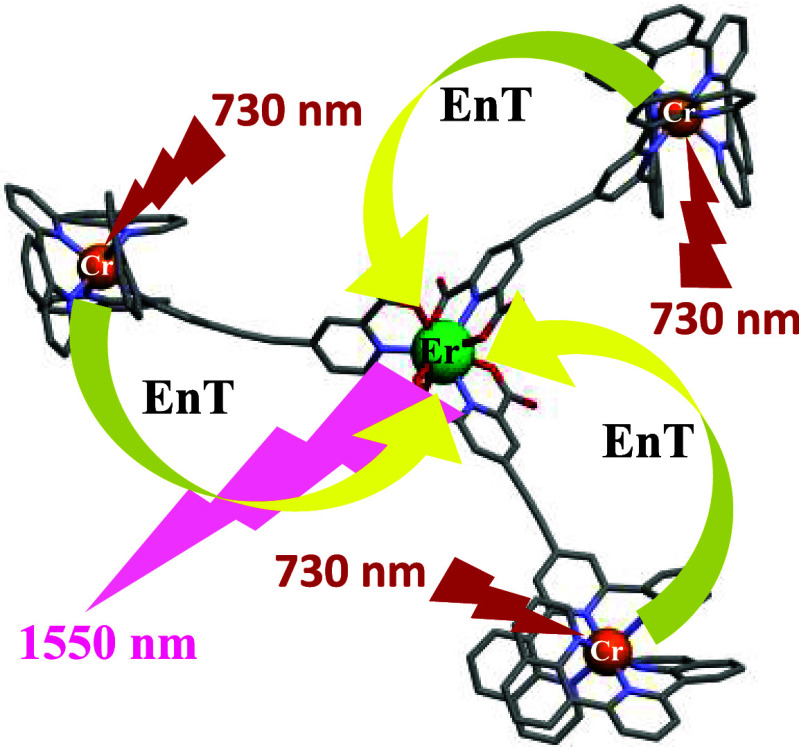

Pseudo-octahedral Cr^III^N_6_ chromophores hold a unique appeal for low-energy sensitization of NIR lanthanide luminescence due to their exceptionally long-lived spin-flip excited states. This allure persists despite the obstacles and complexities involved in integrating both elements into a metallosupramolecular assembly. In this work, we have designed a structurally optimized heteroleptic Cr^III^ building block capable of binding rare earths. Following a complex-as-ligand synthetic strategy, two heterometallic supramolecular assemblies, in which three peripherical Cr^III^ sensitizers coordinated through a molecular wire to a central Er^III^ or Y^III^, have been prepared. Upon excitation of the Cr^III^ spin-flip states, the downshifted Er(^4^I_13/2_ → ^4^I_15/2_) emission at 1550 nm was induced through intramolecular energy transfer. Time-resolved experiments at room temperature reveal a Cr^III^ → Er^III^ energy transfer of 62–73% efficiencies with rate constants of about 8.5 × 10^5^ s^–1^ despite the long donor–acceptor distance (circa 14 Å). This efficient directional intermetallic energy transfer can be rationalized using the Dexter formalism, which is promoted by a rigid linear electron-rich alkyne bridge that acts as a molecular wire connecting the Cr^III^ and Er^III^ ions.

## Abstract 2

The remarkable kinetic inertness of trivalent chromium cations allows for the stepwise preparation of the heteroleptic [Crdqp(H2-L1)]3+ building block which can work as “complex-as-ligand” for the self-assembly of dimetallic [(dqpCrL1)3Er]6+ under basic conditions. The latter supramolecular architecture collects red light (730 nm) for its downshifting toward the infrared domain (1550 nm).

## Introduction

**Figure 1:**
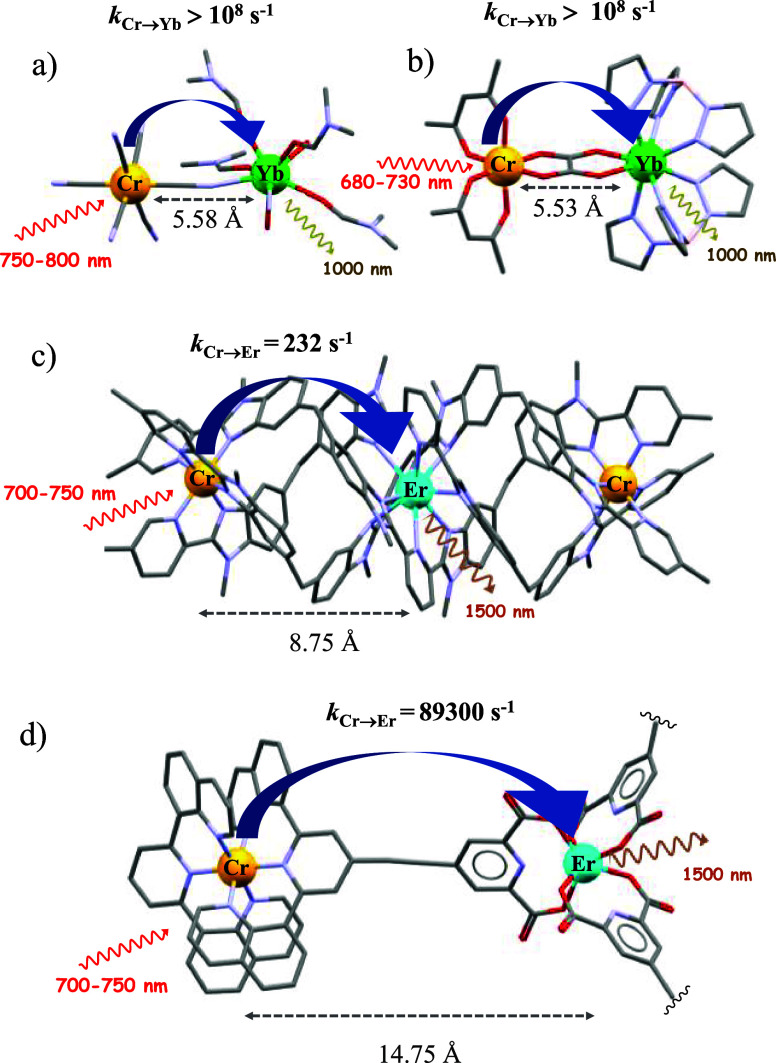
Molecular structures of some assemblies containing Cr–Ln moieties with their respective energy transfer rate constants controlling NIR lanthanide downshifted emission; hydrogen atoms and counterions omitted for clarity. Color codes: Cr (orange), N (blue), C (gray), O (red), Yb (green), and Er (blue). (a) {[Cr(CN)_4_(μ-CN)_2_Yb(H_2_O)_2_(dmf)_4_]}_∞_,^33^ (b) [(**acac**)_2_Cr(**ox**)Yb(**HBpz**_3_)_2_],^24^ (c) [CrErCr(**dipy-pybzimpy**)_3_]^9+^,^29^ and (d) [(**dqp**Cr**L1**)_3_Er]^6+^ reported in this work.

Luminescent lanthanide ions have a wide range of interest within the scientific community due to their applications in biomolecule detection, as nanoprobes, in medical diagnosis, and in telecommunication, lighting, and energy conversion.^1−6^ Some of the trivalent lanthanides exhibit NIR emission, which can be slightly modulated by their chemical environments, and significant endeavors have been devoted to the creation of materials possessing these highly effective optical properties.^7^ In the NIR region, trivalent neodymium (Nd^III^, 9400 cm^–1^), thulium (Tm^III^, 5000–5500 cm^–1^), ytterbium (Yb^III^, 10,000 cm^–1^), and erbium (Er^III^, 6250–6600 cm^–1^) are the most promising in terms of light emission.^8^ Due to the parity-forbidden character of the f-f transitions, direct excitation of the lanthanide ions is very inefficient. The use of closed-shell organic ligands has been thus widely used for sensitization of NIR Ln^III^ luminescence through the so-called antenna effect.^9^ This energy transfer (EnT), operating through Dexter-type double electron exchange, is achievable through the coordination of a ligand to the metal along with a short distance separating them. Nevertheless, when long wavelength light (VIS or NIR) is used to sensitize near-IR/IR luminescent lanthanides (i.e., Nd^III^, Yb^III^, or Er^III^), d-block chromophores acting as energy harvesters are particularly appealing due to their (i) usually long-lived excited state, which ensures more time for inducing the energy transfer to the f-block metal ion, (ii) customizable emission and absorption energies, (iii) limited sensitivity to photobleaching, and (iv) efficient intermetallic communication via directional energy transfer. With this in mind, several polymetallic edifices exploiting precious and inert 4d (Ru^II^ and Pd^II^) and 5d (Os^II^, Ir^III^, and Pt^II^) and their long-lived ^3^MLCT have been proved to sensitize quite efficiently the NIR Ln^III^ luminescence.^10−18^ However, the low abundance and high cost of these transition metals represent important drawbacks for the development of cost-effective energy-converting materials. Within the 3d series, the earth-abundant Cr^III^, embedded within a pseudo-octahedral ligand field, was demonstrated to be an attractive sensitizer, which benefits from (i) favorable inertness and (ii) low cost and long-lived excited states.^19−22^ However, synthetic pathways able to combine several chromium-polypyridyl units and Ln^III^ within an energy converter single (supra)molecule are scarce and dominated by serendipitous synthetic methods.^23−35^ Mostly, d-f arrays incorporating cyanido-bridged Cr^III^–CN–Ln^III^ moieties such as {[Cr(CN)_4_(μ-CN)_2_Ln(H_2_O)_2_(dmf)_4_]}_∞_ (dmf = dimethylformamide), where the Ln = Yb^III^ or Nd^III^ has been described by Ward and co-workers.^33^ In these systems, Cr^III^ acts as a sensitizer for the NIR lanthanide luminescence via a Dexter EnT mechanism with an impressive rate constant *k*_Cr→Ln_ = 10^8^ s^–1^ (Figure 1a). Kaizaki and co-workers also described very efficient short distance EnT via oxalate bridging units in [(acac)_2_Cr(ox)Ln(HBpz_3_)_2_] (Ln = Nd^III^, Yb^III^ or Er^III^; acac = acetylacetonate; HBpz_3_ = tris(pyrazol-1-yl)borate, Figure 1b).^24^ In both cases and at low temperature, only partial EnT occurs, whereas at room temperature a close-to-quantitative thermally activated EnT was observed providing only Ln-centered emission. Long distance intermetallic downshifting phenomena between Cr^III^ (sensitizer) and Ln^III^ (activators: Yb^III^ or Nd^III^) have also been established in triple-stranded helicates with a simplified chemical formula [CrLn(**py-bzimpy**)_3_]^6+^ and [CrLnCr(**dipy-pybzimpy**)_3_]^9+^.^25^ In these systems, long-range electron dipole–dipole formalism (Förster mechanism) was applied for analyzing the rate constants of the energy transfer and the measured *k*_Cr→Ln_ amounts to 232–456 s^–1^,^29−32^ The longer distance between the sensitizer and the activator and their poor orbital overlap are responsible for the much lower intramolecular EnT rate constants (*k*_Cr→Ln_ = 10^2^–10^3^ s^–1^) compared to the electronic communication mediated by short molecular bridges such as oxalate and cyanide (*k*_Cr→Ln_ = 10^7^–10^8^ s^–1^). Under a slow energy transfer regime, the total observed decay rate constant of the excited level of the donor (sensitizer) *k*_**obs**_^**Donor**^ = *k*_**relax**_^**D**^**+***k*_**D** → **A**_^**EnT**^ may be largely dominated by the decay rate constant of the acceptor (luminophore, *k*_**obs**_^**Acceptor**^*≫ k*_**obs**_^**Donor**^) and the emission lifetime of the acceptor mirror that of the donor. This situation occurs for the above-mentioned triple helicates, where the electronic communication between the chromium donor and the lanthanide acceptor is limited and the final NIR Nd^III^ and Yb^III^-based emission, usually displaying microsecond lifetimes, reaches the millisecond regime characteristic of the long-lived Cr^III^ donors (Figure 1c).^29^ Moreover, in these helicates, selective intense irradiation of Cr^III^ produces an energy transfer upconversion (ETU) with the detection of the Er(^4^S_3/2_ → ^4^I_15/2_) green luminescence at 543 nm (18,400 cm^–1^) following near-infrared excitation. Finally, and despite the long distance between the Yb^3+^ and Cr^3+^ (circa 9 Å) and the nonbonding between the two complexes in the crystalline chromium–ytterbium ionic salt of general formula [Yb(**dpa**)_3_][Cr(ddpd)_2_] (dpa = dipicolinate, ddpd = *N*,*N*′- dimethyl-*N*,*N*′-dipyridin-2-ylpyridine-2,6-diamine), a non-negligible Cr → Yb EnT could be detected in the solid state.^36^ Interestingly, Cr^III^ upconversion was also observed upon pumping Yb^III^ centers through a cooperative upconversion (CU) mechanism.^36^

Apart from these remarkable examples, a complex-as-ligand strategy has been tentatively applied by our group to construct 3D-based polymetallic assemblies,^37,38^ but the construction of 3d-4f assemblies remains underexplored. In this work, we report on the synthesis and characterization of a novel heteroleptic Cr^III^ building block, [Cr(**dqp**)(H_2_-**L1**)]^3+^, where **dqp** is 2,6-di(quinolin-8-yl)pyridine and H_2_-**L1** contains a dqp unit linked through an alkyne bridge to a dipicolinic acid. Reaction between the building block and a lanthanide ion Er^3+^ or Y^3+^ led to the formation of a heterotetrametallic assembly (Figure 1d). The full structural, solution, and photophysical analysis of the assembly is analyzed and reported here.

## Results and Discussion

### Synthesis and Characterization of the Ditopic Ligand **10**

**Figure 2:**
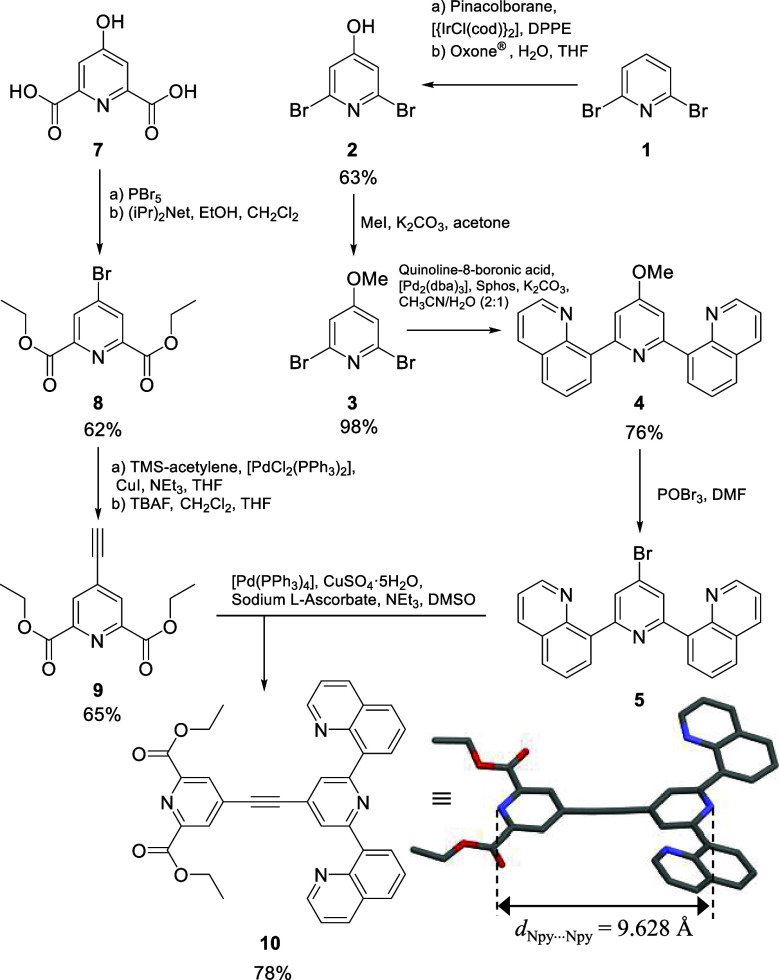
Multistep synthetic scheme for preparing the ditopic ligand diethyl 4-((2,6-di(quinolin-8-yl)pyridin-4-yl)ethynyl)pyridine-2,6-dicarboxylate (**10**). Its molecular structure, as found in the crystal structure, is highlighted; color codes: N (blue), C (gray), and O (red).

The synthesis of the ditopic ligand, **10**, was achieved following the multistep scheme depicted in Figure 2 (see Appendix 1 in the Supporting Information for synthetic details). Starting with 2,6-dibromopyridine (**1**), an iridium-catalyzed activation followed by borylation and subsequent oxidation of the carbon–boron bond with the triple salt *oxone* (**2** KHSO_5_·KHSO_4_·K_2_SO_4_) afforded 2,6-dibromopyridin-4-ol (**2**).^39^ Straightforward protection of the alcohol with methyl iodide yielded 2,6-dibromo-4-methoxypyridine (**3**) that was further combined with quinoline-8-boronic acid in a Suzuki coupling under microwave conditions to yield **4**.^40^ Once **4** was isolated, the methoxy group was replaced by a – Br group,^41,42^ giving **dqp**-Br (**5**). Separately, 8,8′-(4-ethynylpyridine-2,6-diyl)diquinoline (**6**) was synthesized from **5** by Sonogashira cross-coupling.^43,44^ The dipicolinate-moiety was synthesized starting from chelidamic acid (**7**), which was reacted with PBr_5_ to yield diethyl 4-bromopyridine-2,6-dicarboxylate (**8**).^45^ After Sonogashira cross-coupling, diethyl 4-ethynylpyridine-2,6-dicarboxylate (**9**) was achieved and could then be coupled with **5** using a second Sonogashira coupling reaction to yield **10** as supported by ^1^H NMR spectroscopy (Figure S1).

Slow evaporation of a concentrated ethyl acetate solution containing the ligand yielded single crystals of **10** suitable for X-ray diffraction (XRD) (Figures 2, S2, S3, and Tables S1–S3). The nitrogen atoms of each of the two quinolines from the **dqp** moiety are facing the opposite direction as the central nitrogen atom on the pyridine (*transoid* arrangement of the terimine N^∩^N^∩^N ligand, Figures 2 and S2). The two quinoline arms are slightly out of the plane of the central pyridine (19.6° and 31.6°) because of the steric crowding implying the hydrogen atoms on the central pyridine and those of the connected appended quinoline rings that prevent perfect planarity (Figure S3). As a consequence of the triple bond located in the middle of the molecule extending π-delocalization, the two connected pyridine rings are almost coplanar (dihedral angle = 7.65°). The distance between the two coordinating nitrogen atoms on each central pyridine amounts to 9.628(3) Å, which allows for a first rough estimation of Cr–Ln distances expected in the target final assemblies (*d*_Cr-Ln_ ∼ 0.2(Cr–N bond) + 0.96 (N_py_···N_py_) + 0.25 (N–Ln bond) ≈ 1.4 nm).

### Synthesis, Structure, and Spectroscopic Properties of Heteroleptic Complex-as-ligand [Cr(**dqp**)(H_2_-**L1**)](CF_3_SO_3_)_3_

**Figure 3:**
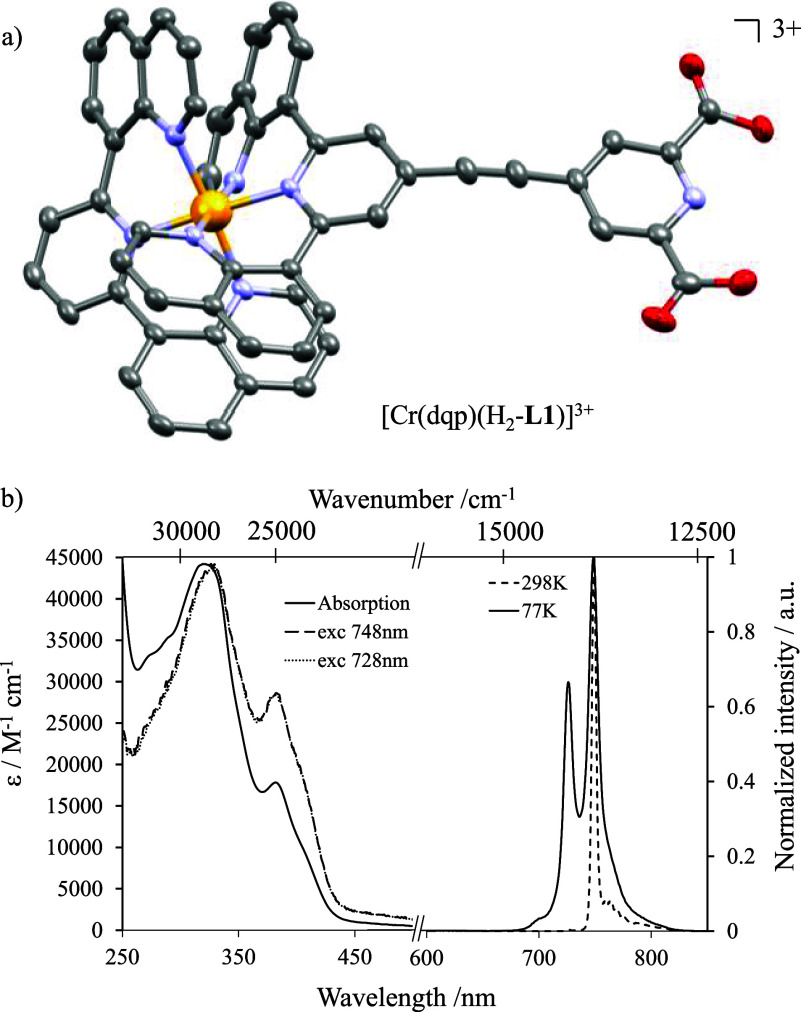
(a) Molecular structure of the cationic complex as found in the crystal structure of [Cr(**dqp**)(H_2_-**L1**)](SO_3_CF_3_)_3_·3.5H_2_O. Ellipsoids are plotted at the 50% probability level, and hydrogen atoms and counterions are omitted for clarity (CCDC 2361449.). Color codes: Cr (orange), N (blue), C (gray), and O (red). (b) Emission, absorption, and excitation spectra of [Cr(**dqp**)(H_2_-**L1**)]^3+^ in acetonitrile solution at room temperature.

The heteroleptic [Cr(**dqp**)(H_2_-**L1**)]^3+^ complex (**L1**^2–^ = 4-((2,6-di(quinolin-8-yl)pyridin-4-yl)ethynyl)pyridine-2,6-dicarboxylate) was prepared by following a previous synthetic strategy described by our group (Figure 3a and Appendix 1 in the Supporting Information for synthetic details).^46,47^ First, the neutral halogeno-complex, [Cr(**10**)Cl_3_], could be obtained by reacting commercially available Cr(THF)_3_Cl_3_ with the tridentate ligand **10** in isopropanol under microwave conditions at 140 °C. The green compound was isolated, washed, and characterized. The addition of 3 equiv of AgSO_3_CF_3_ in acetonitrile led to the soluble intermediate [Cr(**10**)(SO_3_CF_3_)_3_]. While exploiting the known lability of the Cr-OSO_2_CF_3_ bond,^48^ 1 eq. of **dqp** ligand was added to the mixture and after heating under microwave irradiation, the heteroleptic [Cr(**dqp**)(**10**)](SO_3_CF_3_)_3_ complex was obtained (see Appendix 1 in the Supporting Information for details). Upon solubilization of [Cr(**dqp**)(**10**)]^3+^ in water, both ethyl esters could be carefully hydrolyzed “on-the-complex” with an excess of sodium hydroxide that was neutralized after the reaction with HSO_3_CF_3_ leading to [Cr(**dqp**)(H_2_-**L1**)]^3+^. The mass spectrum of the complex displays specific peaks corresponding to [Cr(**dqp**)(H_2_-**L1**)](SO_3_CF_3_)_2_]^+^ and [Cr(**dqp**)(H_2_-**L1**)](SO_3_CF_3_)]^2+^ centered at *m*/*z* = 1205.105 and 528.075 Da, respectively (Figure S4 and Table S4). Upon slow evaporation of a concentrated solution of [Cr(**dqp**)(H_2_-**L1**)]^3+^ in water, monocrystals of [Cr(**dqp**)(H_2_-**L1**)](SO_3_CF_3_)_3_·3.5H_2_O suitable for X-ray diffraction were obtained (Figures 3, S5–S7, Tables S5, and S6). The Cr–N bond lengths are similar to those found in related polypyridyl Cr^III^ complexes, and the N(terminal)–Cr-N(terminal) bite angles of 176.36(8)° (Tables S5 and S6) are in agreement with the values obtained for the parent homoleptic [Cr(**dqp**)_2_]^3+^ complex and its analogues.^49,50^ Similar to the latter complexes, π-stacking between the **dqp** and H_2_-**L1** bound ligands can be appreciated by the relatively short distance between the two respective quinoline centroids (*d*_**dqp**–**L1**_^1^=3.351 Å and *d*_**dqp**–**L1**_^2^=3.386 Å) (Figure S6). The minor interplanar angles between the quinoline rings of **dqp** and **L1** ligands (17.78° and 19.28°) comfort the concept of intramolecular interligand π-stacking as a non-negligible driving force for the complexation process (Figure S7). The Cr···N_py_(dipicolinate) distance amounts to 11.465(3) Å, which allows to refine and confirm the estimation of Cr–Ln distances expected in the target final assemblies to *d*_Cr-Ln_ = 1.1465 + 0.25 (N–Ln bond) = 1.4 nm. Interestingly, the terminal tridentate O^∩^N^∩^O dipicolinic acid binding unit grants the possibility to deprotonate the oxygen atoms, resulting in a doubly negatively charge site, entirely in favor of the complexation of a triply positively charged Ln^III^ ion.

The absorption spectrum of [Cr(**dqp**)(H_2_-**L1**)]^3+^ in acetonitrile shows maxima between 280 and 350 nm (ε > 10^3^ M^–1^ cm^–1^), which can be attributed to standard intense π* ← π transitions, together with bands of lower intensities between 350 and 500 nm (ε < 103 M^–1^ cm^–1^) assigned to mixed metal-centered and ligand-to-metal charge transfer (MC)/LMCT transitions (Figure 3b). The weak transitions located at even lower energies (500–600 nm) are ascribed to the spin-forbidden ^3^π* ← π transitions (Figure 3b). According to time-dependent density functional theory (TD-DFT) calculations on the parent [Cr(**dqp**)_2_]^3+^ compound,^48^ the shoulder at 401 nm can be ascribed to the ligand field transition Cr(^4^T_2_ ← ^4^A_2_), leading to an estimated ligand field splitting of 25,000 cm^–1^. Upon excitation at 350 nm, within the ligand-centered transitions, [Cr(**dqp**)(H_2_-**L1**)]^3+^ displays a narrow dual emission with maxima at 730 nm (13,698 cm^–1^) and 755 nm (13,245 cm^–1^) assigned to the spin-flip Cr(^2^E,^2^T_1_ → ^4^A_2_) transitions (Figure 3b). The emission band maxima are slightly red-shifted by 150 cm^–1^ compared to the parent [Cr(**dqp**)_2_]^3+^ complex. This drift can be reasonably assigned to a larger nephelauxetic effect produced by improved electronic delocalization onto the extended H_2_-**L1** ligand in the heteroleptic analogue [Cr(**dqp**)(H_2_-**L1**)]^3+^.^51^ The measured quantum yields for the latter complex amount to 16 and 0.3% in acetonitrile at room temperature under anaerobic and aerobic conditions, respectively, which are among the highest values reported for Cr(III) complexes.^52,53^ The two spin-flip transitions share the same lifetime of about 1.62(6) ms in deaerated acetonitrile solution, demonstrating thermally equilibrated states at room temperature (Figure S13). At 77 K, the high-energy emission band vanishes, and only the lowest low-lying microstate is populated and detectable together with its vibrational progression. The low temperature excited state lifetime amounts to 2.63(7) ms in frozen H_2_O/DMSO (1:1) solution (Figure S14). The excitation spectra recorded upon monitoring of the emission bands at 748 and 728 nm match the absorption spectrum, demonstrating the participation of π–π*, LMCT, and LMCT/MC excited states for feeding the emissive Cr-centered doublet states (Figure 3b).

### Synthesis and Solution Studies of the [(dqpCrL1)_3_Ln]^6+^ (Ln = Er^3+^ and Y^3+^) Assemblies

**Figure 4:**
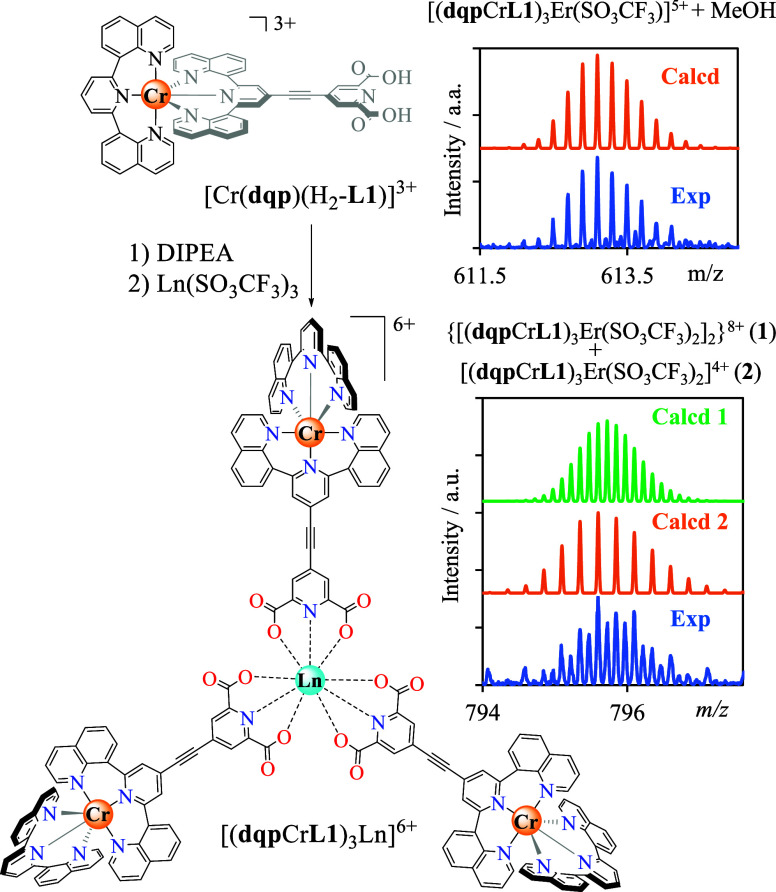
Synthesis of [(**dqp**Cr**L1**)_3_Ln]^6+^ assemblies from [Cr(**dqp**)(H_2_-**L1**)]^3+^ and associated HR-ES/MS spectra.

The supramolecular [(**dqp**Cr**L1**)_3_Ln](SO_3_CF_3_)_6_ assemblies (Ln = Er^3+^ or Y^3+^) are obtained by reacting the doubly deprotonated heteroleptic complex-as-ligand [**dqp**Cr**L1**]^+^, obtained via the reaction of 1 eq. of [Cr(**dqp**)(H_2_-**L1**)]^3+^ in the presence of 2 eq. of *N*,*N*-diisopropylethylamine, with stoichiometric amount (3:1) of the lanthanide triflate salt in acetonitrile at room temperature (Figure 4a and Appendix 1 in the Supporting Information for synthetic details). Slow precipitation induced by the diffusion of diethyl ether provides an orange precipitate that was filtered, washed with diethyl ether, and dried. Despite countless crystallization attempts, single crystals suitable for X-ray diffraction were not obtained. However, the spectrophotometric titration combined with speciation experiments and ESI–MS spectra support the formation and stability of the multimetallic [(**dqp**Cr**L1**)_3_Er]^6+^ and [(**dqp**Cr**L1**)_3_Y]^6+^ assemblies in solution. The mass spectra of the [(**dqp**Cr**L1**)_3_Ln]^6+^ (Ln = Er or Y) display a similar pattern and show excellent agreement with the theoretical isotopic distributions for the molecular ion (Figures 4, S8–S11, Tables S7, and S8). It is worth stressing here that the final assemblies are prone to dimerization in the gas phase, probably as a result of the presence of triflate counteranions, which are able to link two cationic units via their accessible and shared central lanthanide cations (Figure 4 bottom).

The complexation and stability constants of the complex-as-ligand [**dqp**Cr**L1**]^+^ with a trivalent lanthanide ion were analyzed by spectrophotometric titrations because of the incompatibility of the slow electronic relaxation of Cr(III) complexes with high-resolution NMR techniques. [**dqp**Cr**L1**]^+^ was stepwise titrated with Ln(SO_3_CF_3_)_3_ (Ln = Er^3+^ or Y^3+^) in dry acetonitrile. After each addition, a timeout of 1 min was carried out to ensure that thermodynamic equilibrium was reached. At each point, the absorption spectrum of the solution was recorded. After data treatment based on evolving factor analysis as implemented in the software ReactLab Equilibria,^54−57^ two sets of three stability constants, corresponding to the formation of 1:1, 1:2, and 1:3 complexes, could be extracted (eqs 1–3).
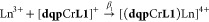
1

with log(β_1_) = 9.177 ± 0.016 for Ln = Er^3+^ and log(β_1_) = 7.467 ± 0.005 for Ln = Y^3+^
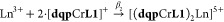
2

with log(β_2_) = 16.912 ± 0.031for Ln = Er^3+^ and log(β_2_) = 13.888 ± 0.009 for Ln = Y^3+^
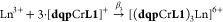
3

with log(β_3_) = 23.403 ± 0.045 for Ln = Er^3+^ and log(β_3_) = 19.386 ± 0.012 for Ln = Y^3+^.

**Figure 5:**
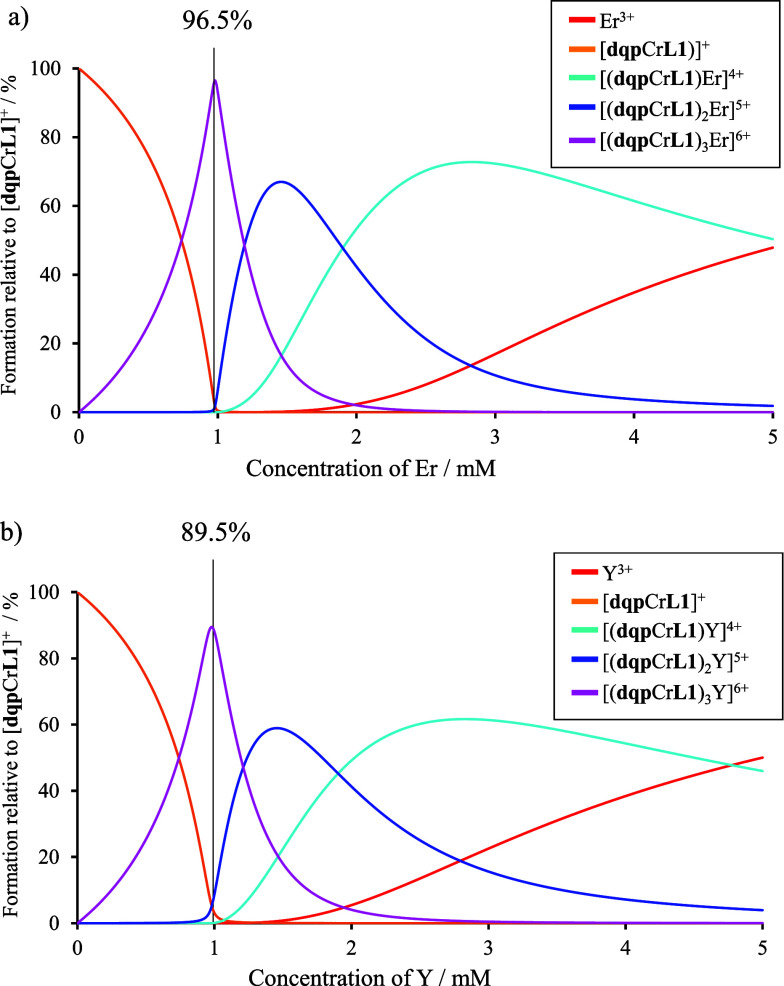
Simulated speciations relative to [**dqp**Cr**L1**]^+^ and using the formation constants pertinent to eqs 1–3. (a) Titration of a 3 mM solution of [**dqp**Cr**L1**]^+^ with Er(SO_3_CF_3_)_3_ in CH_3_CN. (*c*_[(**dqp**Cr**L1**)_3_Er]_ = 1 mM) and (b) simulation of a titration of a 3 mM solution of [**dqp**Cr**L1**]^+^ with Y(SO_3_CF_3_)_3_ in CH_3_CN (*c*_[(**dqp**Cr**L1**)_3_Y]_ = 1 mM).

The significant increases of the formation constants with decreasing effective ionic radii in going from Y^3+^ to Er^3+^ are in line with the standard electrostatic trend reported for [Ln(dipicolinate)_*n*_]^(3–2*n*)+^ (*n* = 1–3) along the lanthanide series.^58,59^ A simulation of the speciation in solution using HySS software^60^ for a target 1 mM solution of [(**dqp**Cr**L1**)_3_Ln]^6+^, i.e., the concentration at which the photophysical experiments were run, shows that 96.5% of the complex-as-ligand is involved in the [(**dqp**Cr**L1**)_3_Er]^6+^ and 89.5% in [(**dqp**Cr**L1**)_3_Y]^6+^ (Figure 5).

### Absorption, Emission, and Energy Transfer Properties of the [(dqpCrL1)_3_Ln]^6+^ Assemblies

**Figure 6:**
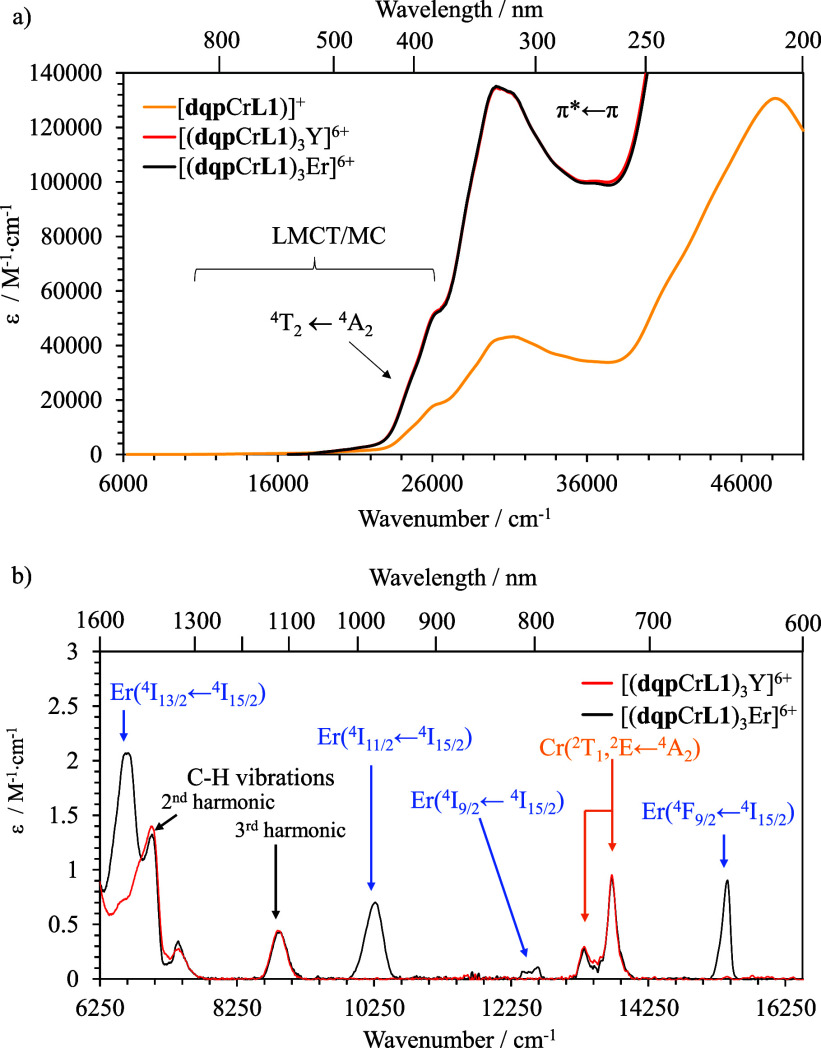
(a) UV absorption spectra recorded in acetonitrile at 1 mM for [(**dqp**Cr**L1**)_3_Er]^6+^ (black trace), [(**dqp**Cr**L1**)_3_Y]^6+^ (red trace) and [**dqp**Cr(**L1**)]^+^ (yellow trace) in a 0.2 mm cuvette. (b) Absorption spectra of the tetra-metallic species in the (near) infrared region of the electromagnetic spectrum at 1 mM in acetonitrile.

The UV absorption spectra of the assemblies were recorded at the millimolar concentration using a 0.2 mm path length cuvette (Figure 6). As expected, the total ε values of the d-f complexes are 3 times larger than those of the deprotonated [**dqp**Cr(**L1**)]^+^ since they involve three such units in their molecular formula (Figure 6a). The UV region is dominated by the presence of π* ← π ligand-centered transitions of the organic backbone, whereas the transitions at lower energies in the 300–400 nm range (27,000 and 20,000 cm^–1^ respectively) are attributed to a mixture of ligand-to-metal charge transfers (LMCT), metal-centered (MC), and a fraction of metal-to-ligand charge transfers (MLCT), similar to the isolated [Cr(**dqp**)(H_2_**-L1**)]^3+^ precursor (Figure 3b). Upon recording the absorption spectra in the near-infrared region (NIR, up to 1600 nm) in concentrated acetonitrile solutions, the parity-forbidden f-f and parity- and spin-forbidden d-d transitions were detected (Figure 6b). The radiative rate constant (*k*_rad_) for the d-d and f-f transitions in the assemblies was calculated by the integration of the band absorptions and following the Strickler–Berg eq 4.^61,62^
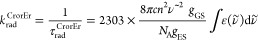
4

in which, *n* is the refractive index (*n*_CH3CN_ = 1.33), ν̃ is the barycenter of the transition, and *N*_A_ is Avogadro’s number. The two terms *g*_ES_ and *g*_GS_ are the degeneracy of the excited state and the ground state levels, respectively. In the case of the trivalent chromium atom, *g*_GS_ = 4 and *g*_ES_ = 4 for the ^2^E level and *g*_ES_ = 6 for the ^2^T_1_ level. For the trivalent erbium atom, *g*_GS_ = (2*J* + 1) and *g*_ES_ = (2*J*′ + 1). Finally, ∫ε(ν̃)dν̃ is the integral of the absorption band (in cm^2^). The computed values are gathered in Table 1. In both [(**dqp**Cr**L1**)_3_Er]^6+^ and [(**dqp**Cr**L1**)_3_Y]^6+^, the *k*_rad_^Cr^ for the spin-flip transitions are in line with the parent [Cr(**dqp**)_2_]^3+^ compound.^49^

**Table 1:** Photophysical Parameters of the Assemblies [(**dqp**Cr**L1**)_3_Ln]^6+^ (Ln = Y, Er)

	excited level	ν̃/cm^–1^	ε_max_/M^–1^cm^–1^	τ_rad_^Cr_3_Ln^/ms^a^	τ_obs_ (μs)^b^		ϕ (%)^c^^,^^d^	ϕ (%)^d^^,^^e^
[(**dqp**Cr**L1**)_3_Y]^6+^	Cr(^2^E)	13,738	0.95	34.79	20.90^f^	31.24	0.3	16
	Cr(^2^T_1_)	13,371	0.26	78.54				
	Cr(^2^E)	13,738	0.95	32.01	7.90^f^	8.2^g^	0.3	16
	Cr(^2^T_1_)	13,371	0.27	45.08				
	Er(^4^I_13/2_)	6607	2.06	8.84	5.0^f^	9.85^g^	0.063^h^	
[(**dqp**Cr**L1**)_3_Er]^6+^	Er(^4^I_11/2_)	10,248	0.66	6.33				
	Er(^4^I_9/2_)	12,544	0.09	21.80				
	Er(^4^F_9/2_)	15,356	0.88	2.58				

a τ_rad_ = 1/*k*_rad_.

b τ_obs_ from time-resolved experiments at 293 K.

c In aerobic conditions.

d λ_exc_ = 435 nm, using [Cr(ddpd)_2_]^3+^ as a reference.

e In anaerobic conditions. Lifetime: estimated relative uncertainty ±10%.

f In acetonitrile solution.

g In the solid state.

h Calculated with eq 7.

**Figure 7:**
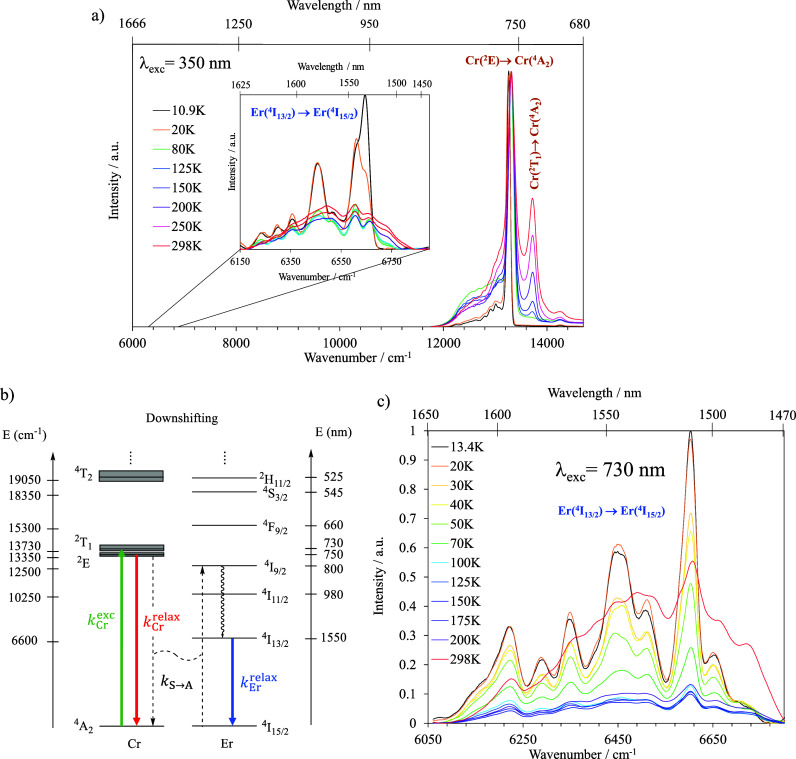
(a) Variable temperature emission measurement of Cr(^2^T_1_ → ^4^A_2_), Cr(^2^E → ^4^A_2_) and Er(^4^I_13/2_ → ^4^I_15/2_) in [(**dqp**Cr**L1**)_3_Er]^6+^ in the solid state with λ_exc_ = 350 nm. (b) Jablonski diagram for a downshifting process operating in molecular [(**dqp**Cr**L1**)_3_Er]^6+^. (c) Variable temperature emission measurement of in [(**dqp**Cr**L1**)_3_Er]^6+^ in the solid state with λ_exc_ = 730 nm.

Upon excitation of the tetra-metallic assemblies at 350 nm, the typical emission bands Cr(^2^T_1_ → ^4^A_2_), Cr(^2^E → ^4^A_2_) in both [(**dqp**Cr**L1**)_3_Y]^6+^ and [(**dqp**Cr**L1**)_3_Er]^6+^, as well as Er(^4^I_13/2_ → ^4^I_15/2_) in the latter complex, have been recorded in the solid state and solution at variable temperatures (10–298 K). In both systems, the room temperature Cr-based dual emission observed at 730 nm (13,730 cm^–1^) and 755 nm (13,350 cm^–1^) slowly evolves toward a single emission from the Cr(^2^E) level at low temperature (Figure 7a). It can be reasonably assumed that the ^2^T_1_ level starts to be populated from 90 K upward, preventing any thermal population through internal conversion between the ^2^T_1_ and the ^2^E level at lower temperature. For [(**dqp**Cr**L1**)_3_Er]^6+^, the broad and structured emission located at 1500–1600 nm corresponds to the characteristic Er(^4^I_13/2_ → ^4^I_15/2_) transition. Upon decreasing the temperature, the low-lying microstate is populated as a consequence of a thermal equilibration at 10 K (Figure 7a). As confirmed, each emissive transition’s excitation spectra match the absorption spectra of the corresponding compound (Figure S12).

To investigate the electronic communication between the Cr^III^ and Er^III^ ions, steady-state and time-resolved experiments were carried out. For this purpose, a continuous-wave laser at 730 nm was used to hit the Cr(^2^T_1_ ← ^4^A_2_) spin-flip band, thus bringing the 3d metal center into its Cr(^2^T_1_) excited state. If EnT takes place, the *quasi-*isoenergetic Er(^4^I_9/2_) level will be populated to quickly relax to the long-lived Er(^4^I_13/2_) energy level, from which the typical NIR emission of the erbium might be observed (Figure 7b). The evolution of the emission from the Er(^4^I_13/2_) level upon spin-flip excitation (at 730 nm) was observed at different temperatures indicating a successful Cr→Er light-downshiting (Figure 7 b,c). The efficiency of the intermetallic energy transfer is related to the excited state lifetimes of the donor Cr^3+^ in the presence of the acceptor in [(**dqp**Cr**L1**)_3_Er]^6+^, compared with that in the absence of acceptor as measured in [(**dqp**Cr**L1**)_3_Y]^6+^ (eq 5).
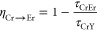
5

In acetonitrile solution at room temperature, the radiative decay is monoexponential and the associated excited state lifetime of the Cr^III^ center drops from 40.7 to 13.8 μs in going from [(**dqp**Cr**L1**)_3_Y]^6+^ to [(**dqp**Cr**L1**)_3_Er]^6+^ (Figures S17 and S18) which result in an EnT efficiency (η_Cr→Er_) of 66%. A similar behavior was found in the solid state, with values of 31.2 and 8.2 μs (Figures S15 and S16), respectively, giving a 73% efficiency for the downshifting energy transfer. The rate of the energy transfer, *k*_Cr→Er_, could be calculated using eq 6
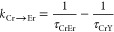
6

from which exceptional values of 47,900 and 89,300 s^–1^ in solution and in the solid state, respectively, were computed at room temperature. This demonstrates the efficient and fast phonon-assisted EnT between the donor and the acceptor, which is mediated by the alkyne bridge despite the long distance (circa 1.4 nm). A Dexter-type mechanism promoted by the alkyne bridge and some non-negligible overlap of the wave function of the excited state of the donor and the acceptor are probably at the origin of such an efficient EnT.

**Figure 8:**
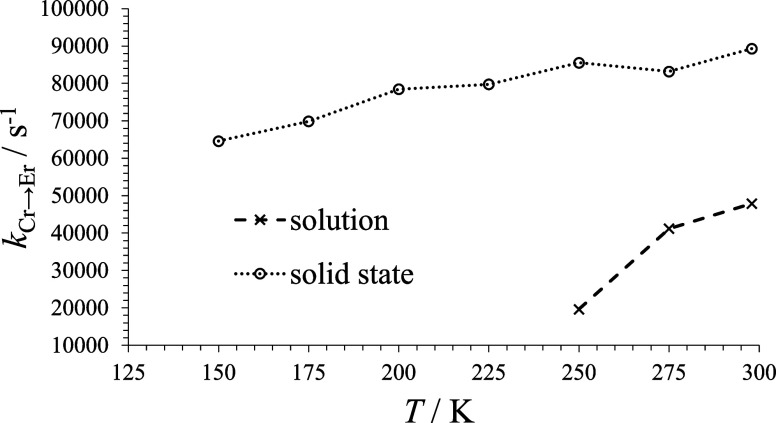
Variation of the EnT rate constant in the [(**dqp**Cr**L1**)_3_Er]^6+^ assembly as a function of the temperature in the solid state and in solution (acetonitrile, 10^–3^ M).

It is noteworthy that as the temperature decreases, both the efficiency (Figure S19) and the rate of energy transfer (Figure 8) decrease in solution and in the solid state. This is in line with a temperature-dependent energy transfer phenomenon, commonly observed in dominant Dexter energy transfer processes.

Finally, the luminescence quantum yield for the emission of Er upon sensitization of the spin-flip band of Cr was calculated to be 0.063% with eq 7
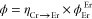
7where the ϕ_Er_^Er^ is the intrinsic quantum yield. The latter parameter reflects the extent of nonradiative deactivation processes occurring through interactions with the surroundings of the metal ion, and it is estimated as the ratio between τ_obs_ and τ_rad_ of [(**dqp**Cr**L1**)_3_Er]^6+^ at 1530 nm (Table 1). Only a few Cr^III^–Er^III^ compounds have been reported in the literature, most of them using cyanide bridges to put the metals in communication.^26−28,32,35,64−67^ The presented [(**dqp**Cr**L1**)_3_Er]^6+^ displays the longest intermetallic distance among them. The kinetic study of the intramolecular energy transfer between Cr^III^ and Er^III^ is even more scarce and carried out in the trimetallic [CrErCr(**dipy-pybzimpy**)_3_]^9+^ (Figure 1c), and in the corresponding bimetallic assembly.^68,69^ The rate constant obtained reaches the 10^2^ s^–1^ range for an intermetallic distance of circa 9.3 Å, which makes the present system 200–400 times faster despite the longer intermetallic distance (circa 14 Å). More largely, only a handful of heterometallic Cr^III^–Ln^III^ molecular assemblies were kinetically studied, and the rate of energy transfer was calculated.^24,27,29,31−33,70,71^ Because the energy rate transfer strongly depends on the overlap integral between the emission spectra of the donor and the absorption spectra of the acceptor, typical values found in the literature for Cr^III^–Nd^III^ and Cr^III^–Eu^III^ are in the 10^3^ s^–1^ range for an intermetallic distance of circa 9.3 Å.^27,29,31,32,70^ In these systems, energy transfer mainly operates through a Förster-type mechanism. To compare the [(**dqp**Cr**L1**)_3_Er]^6+^ with another system operating through Dexter energy transfer, Lazarides et al. studied a cyano-bridged Cr^III^–Yb^III^ system with an intermetallic distance of 5.59 Å.^33^ The rate of energy transfer was estimated to be greater than 1 × 10^8^ s^–1^. The herein reported supramolecular assembly is, to the best of our knowledge, the first reported example of a molecular Cr^III^–Er^III^ complex displaying Dexter-type energy transfer.

## Conclusions

A structurally optimized [Cr(**dqp**)(H_2_-**L1**)]^3+^ building block was designed and characterized. After deprotonation of the dipicolinic acid moiety, the complex was able to bind rare earths (Y^III^ or Er^III^) forming the heterotetrametallic [(**dqp**Cr**L1**)_3_Y]^6+^ and [(**dqp**Cr**L1**)_3_Er]^6+^ supramolecular assemblies. Valuable Er^III^-based downshifting emission at 1550 nm upon irradiation on the spin-flip transition of sensitizer Cr(^2^E,^2^T_1_ ← ^4^A_2_) at 730 nm was extensively studied at different temperatures. Time-resolved experiments showed that the large EnT efficiency and rate constants are dominated by a “Dexter-type” (through-bonds) energy transfer. The intermetallic distance between the two trivalent d and f metals was increased from ∼9.2 Å in triple helices CrEr and CrErCr to ∼14.15 Å in the current system. Despite the longer intermetallic distance, the rate of transfer is increased from 232 to 456 s^–1^ to 89,300 s^–1^ reaching the range reported for 4d-4f and 5d-4f systems, which benefit from the expansion of the d-orbitals accompanying the removal of the primogenic effect.^63^ This is the first example of a d-f heterometallic molecular system using chromium, for which “Dexter-type” energy transfer over a nanometric distance is observed.

## Experimental Section

No uncommon hazards are noted.

### Solvents and Starting Materials

Reagent grade acetonitrile (ACN) was distilled from CaH_2_. All other chemicals were purchased from commercial suppliers and used without further purification. Silica-gel plates (Merck, 60 F254) were used for thin-layer chromatography, and preparative column chromatography was performed using SiliaFlash silica gel P60 (0.04–0.063 mm).

### Spectroscopic and Analytical Measurements

^1^H and NMR spectra were recorded at 298 K on a Bruker Avance 400 MHz spectrometer. Spectrophotometric titrations were performed with a J&M diode array spectrometer (Tidas series) connected to an external computer. Mathematical treatment of the spectrophotometric titrations was performed with factor analysis and with ReactLab Equilibria^A1-A4^ (previously Specfit/32). A pneumatically assisted electrospray (ESI) mass spectrum was recorded on an Applied Biosystems API 150EX LC/MS System equipped with a Turbo Ionspray source. High resolution mass spectra were recorded on a Xevo G2-TOF HRMS instrument equipped with a Zspray Lockspray ESI/APCI/ESCi electrospray by Waters. Elemental analyses were performed by Paglia from the Microchemical Laboratory of the University of Geneva. Solution state absorption spectra were recorded using a Lambda 1050 PerkinElmer spectrometer (quartz cell path length 1 cm, 1 mm or 0.2 mm, 250–1600 nm domain). Solid state absorption spectra were recorded by using a Lambda 900 PerkinElmer spectrometer (using quartz plates). Emission spectra (excitation at 355 nm) and excitation spectra were recorded, with a Fluorolog (Horiba Jobin-Yvon), equipped with iHR320, a xenon lamp 450 W Illuminator (FL-1039*A*/40A), a water-cooled photo multiplier tube (PMT Hamamatsu R2658 or R928) for the 250–850 nm range, and a liquid nitrogen cooled photo multiplier tube (Hamamatsu IR PMT H10330-75) for the 800–1650 nm range. Both detectors are corrected for the spectral response of the system. Emission spectra (excitation at 730 nm) were recorded with an MDL-III-730-1.5W as a light source connected to a PSU-III-LED power supply. Variable temperature measurements were performed using a closed-cycle cryosystem (Janis, CCS-900/204N) with the sample sitting in the exchange gas (helium) to achieve efficient cooling. The samples were put in 2 mm diameter cylindrical quartz cuvettes or between two flat quartz plates. The cuvettes were sealed with fast drying silver agar gel and parafilm to then be mounted on a metallic copper sample holder. For time-resolved experiments, the decay curves were recorded from previously excited samples with a photomultiplier (Hamamatsu R2658 or R928 or Hamamatsu IR PMT H10330-75) and a digital oscilloscope (Tektronix MDO4104C). Pulsed excitation at 355 nm was obtained with the third harmonic of a pulsed Nd:YAG laser (Quantel Qsmart 850).

## Supporting Information Available

Complete experimental details, complex syntheses, characterizations, and photophysical data (PDF)

## References

[ref1] Wu W.; Zhang X.; Kornienko A. Y.; Kumar G. A.; Yu D. C.; Emge T. J.; Riman R. E.; Brennan J. G. Efficient NIR Emission from Nd, Er, and Tm Complexes with Fluorinated Selenolate Ligands. Inorg. Chem. 2018, 57, 1912–1918. 10.1021/acs.inorgchem.7b02814. 29373785

[ref2] Sun G. T.; Xie Y.; Sun L. N.; Zhang H. J. Lanthanide upconversion and downshifting luminescence for biomolecules detection. Nanoscale Horiz. 2021, 6, 766–780. 10.1039/D1NH00299F. 34569585

[ref3] Yang Y. J.; Tu D. T.; Zhang Y. Q.; Zhang P.; Chen X. Y. Recent advances in design of lanthanide-containing NIR-II luminescent nanoprobes. Iscience 2021, 24, 102062 10.1016/j.isci.2021.102062. 33604522 PMC7873658

[ref4] Bünzli J.-C. G. Lanthanide light for biology and medical diagnosis. J. Luminesc. 2016, 170, 866–878. 10.1016/j.jlumin.2015.07.033.

[ref5] Ye H. Q.; Li Z.; Peng Y.; Wang C. C.; Li T. Y.; Zheng Y. X.; Sapelkin A.; Adamopoulos G.; Hernández I.; Wyatt P. B.; Gillin W. P. Organo-erbium systems for optical amplification at telecommunications wavelengths. Nat. Mater. 2014, 13, 382–386. 10.1038/nmat3910. 24651429

[ref6] Bünzli J.-C. G. Applications of Rare Earths in The lanthanides and Actinides, Synthesis, Reactivity, Properties and Applications; Liddle S. T.; Mills D. P.; Natrajan L. S., Eds; World Scientific: London, 2022; Chap. 17, pp 633–686.

[ref7] Bünzli J.-C. G. On the design of highly luminescent lanthanide complexes. Elsevier 2015, 293, 19–47. 10.1016/j.ccr.2014.10.013.

[ref8] Comby S.; Bünzli J.-C. G. Lanthanide near-Infrared Luminescence in Molecular Probes and Devices. In Handbook on the Physics and Chemistry of Rare Earths; GschneidnerJr K. A.; Bünzli J.-C. G.; Pecharsky V. K., Eds; Elsevier Science: Amsterdam, 2007; Vol. 37, pp 217–470.

[ref9] Ward M. D. Mechanisms of sensitization of lanthanide(III)-based luminescence in transition metal/lanthanide and anthracene/lanthanide dyads. Coord. Chem. Rev. 2010, 254, 2634–2642. 10.1016/j.ccr.2009.12.001.

[ref10] Northrop B. H.; Zheng Y.-R.; Chi K.-W.; Stang P. J. Self- Organization in Coordination-Driven Self-Assembly. Acc. Chem. Res. 2009, 42, 1554–1563. 10.1021/ar900077c. 19555073 PMC2764814

[ref11] Chakrabarty R.; Mukherjee P. S.; Stang P. J. Supramolecular Coordination: Self-Assembly of Finite Two- and Three-dimensional Ensembles. Chem. Rev. 2011, 111, 6810–6918. 10.1021/cr200077m. 21863792 PMC3212633

[ref12] Thomas J. A. Metal Ion Directed Self-Assembly of Sensors for Ions Molecules and Biomolecules. Dalton Trans. 2011, 40, 12005–12016. 10.1039/c1dt10876j. 21986864

[ref13] Mede T.; Jäger M.; Schubert U. S. ’Chemistry-on-the- complex’: Functional Ru(II) polypyridyl-type sensitizers as divergent building blocks. Chem. Soc. Rev. 2018, 47, 7577–7627. 10.1039/C8CS00096D. 30246196

[ref14] Datta S.; Saha M. L.; Stang P. J. Hierarchical Assemblies of Supramolecular Coordination Complexes. Acc. Chem. Res. 2018, 51, 2047–2063. 10.1021/acs.accounts.8b00233. 30133252 PMC6348110

[ref15] Agosti A.; Kuna E.; Bergamini G. Divergent terpyridine-Based Coordination for the Construction of Photoactive Supramolecular Structures. Eur. J. Inorg. Chem. 2019, 2019, 577–584. 10.1002/ejic.201801263.

[ref16] Li F.; Lindoy L. F. Metalloligand Strategies for Assembling Heteronuclear Nanocages - Recent Developments. Aust. J. Chem. 2019, 72, 731–741. 10.1071/CH19279.

[ref17] Wang S. C.; Cheng K. Y.; Fu J. H.; Cheng Y. C.; Chan Y. T. Conformational Regulation of Multivalent Terpyridine Ligands for Self-Assembly of Heteroleptic Metallo-Supramolecules. J. Am. Chem. Soc. 2020, 142, 16661–16667. 10.1021/jacs.0c06618. 32881485

[ref18] Balzani V.; Ceroni P.; Credi A.; Venturi M. Ruthenium Tris(bipyridine) Complexes: Interchange Between Photons and Electrons in Molecular-scale Devices and Machines. Coord. Chem. Rev. 2021, 433, 213758 10.1016/j.ccr.2020.213758.

[ref19] Büldt L. A.; Wenger O. S. Chromium complexes for luminescence, solar cells, photoredox catalysis, upconversion, and phototriggered NO release. Chem. Sci. 2017, 8, 7359–7367. 10.1039/C7SC03372A. 29163886 PMC5672834

[ref20] Forster C.; Heinze K. Photophysics and photochemistry with Earth-abundant metals - fundamentals and concepts. Chem. Soc. Rev. 2020, 49, 1057–1070. 10.1039/C9CS00573K. 32025671

[ref21] Wegeberg C.; Wenger O. S. Luminescent First-Row Transition Metal Complexes. JACS Au 2021, 1, 1860–1876. 10.1021/jacsau.1c00353. 34841405 PMC8611671

[ref22] Jimenez J.-R.; Doistau B.; Poncet M.; Piguet C. Heteroleptic Trivalent Chromium in Coordination Chemistry: Novel Building Blocks for Addressing Old Challenges in Multimetallic Luminescent Complexes. Coord. Chem. Rev. 2021, 434, 213750 10.1016/j.ccr.2020.213750.

[ref23] Brayshaw P. A.; Bünzli J.-C. G.; Froidevaux P.; Harrowfield J. M.; Kim Y.; Sobolev A. N. Synthetic, structural and spectroscopic studies on solids containing tris(dipicolinato) rare earth anions and transition or main group metal cations. Inorg. Chem. 1995, 34, 2068–2076. 10.1021/ic00112a019.

[ref24] Sanada T.; Suzuki T.; Yoshida T.; Kaizaki S. heterodinuclear complexes containing d- and f-block elements: synthesis, structural characterization and metal-metal interactions of novel chromium(III)-lanthanide(III) compounds bridged by oxalate. Inorg. Chem. 1998, 37, 4712–4717. 10.1021/ic971568k. 11670625

[ref25] Subhan M. A.; Suzuki T.; Kaizaki S. Stereospecific assembly of chiral Λ-Cr(III)-Δ-Ln(III) oxalato bridged dinuclear 3d-4f complexes (Ln = Yb, Dy) and near infrared dichroism in the 4f-4f transitions. J. Chem. Soc., Dalton Trans. 2001, 492–497. 10.1039/b007369p.

[ref26] Subhan M. A.; Suzuki T.; Kaizaki S. Solution NIR CD and MCD in 4f-4f transitions of a series of chiral 3d-4f dinuclear complexes: X-ray structures of (D-L)-[(acac)2Cr(III)(m-ox)Ln(IIII)(HBpz3)2] (Ln = Sm, Ho, Er). J. Chem. Soc., Dalton Trans. 2002, 1416–1422. 10.1039/b108770c.

[ref27] Cantuel M.; Bernardinelli G.; Imbert D.; Bünzli J.-C. G.; Hopfgartner G.; Piguet C. A kinetically inert and optically active Cr(III) partner in thermodynamically self-assembled heterodimetallic non-covalent d-f podates. J. Chem. Soc., Dalton Trans. 2002, 1929–1940. 10.1039/b200011c.

[ref28] Subhan M. A.; Nakata H.; Suzuki T.; Choi J.-H.; Kaizaki S. Simultaneous observation of low temperature 4f-4f and 3d-3d emission spectra in a series of Cr(III)oxLn(III) assembly. J. of. Luminesc. 2003, 101, 307–315. 10.1016/S0022-2313(02)00573-2.

[ref29] Imbert D.; Cantuel M.; Bünzli J.-C. G.; Bernardinelli G.; Piguet C. Extending lifetimes of lanthanide-based near-infrared emitters (Nd, Yb) in the millisecond range through Cr(III) sensitization in discrete bimetallic edifices. J. Am. Chem. Soc. 2003, 125, 15698–15699. 10.1021/ja0386501. 14677932

[ref30] Cantuel M.; Bernardinelli G.; Muller G.; Riehl J. P.; Piguet C. The first enantiomerically pure helical noncovalent tripod for assembling nine-coordinate lanthanide(III) podates. Inorg. Chem. 2004, 43, 1840–1849. 10.1021/ic035292u. 15018502

[ref31] Torelli S.; Imbert D.; Cantuel M.; Bernardinelli G.; Delahaye S.; Hauser A.; Bünzli J.-C. G.; Piguet C. Tuning the Decay Time of Lanthanide-Based Near Infrared Luminescence from Micro- to Milliseconds through d-f Energy Transfer in Discrete Heterobimetallic Complexes. Chem.—Eur. J. 2005, 11, 3228–3242. 10.1002/chem.200401158. 15779094

[ref32] Cantuel M.; Gumy F.; Bünzli J.-C. G.; Piguet C. Encapsulation of labile trivalent lanthanides into a homobimetallic chromium(III)-containing triple-stranded helicate. Synthesis, Characterization, and divergent intramolecular energy transfers. Dalton Trans. 2006, 2647–2660. 10.1039/B602392D. 16804577

[ref33] Lazarides T.; Davies G. M.; Adams H.; Sabatini C.; Barigelletti F.; Barbieri A.; Pope S. J. A.; Faulkner S.; Ward M. D. Ligand-field excited states of hexacyanochromate and hexacyanocobaltate as sensitisers for near-infrared luminescence from Nd(III) and Yb(III) in cyanide-bridged d–f assemblies. Photochem. Photobiol. Sci. 2007, 6, 1152–1157. 10.1039/b708683k. 17973046

[ref34] Xu H.-B.; Li J.; Zhang L. Y.; Huang X.; Li B.; Chen Z.-N. Structures and photophysical properties of homo- and heteronuclear lanthanide(III) complexes with bridging 2-methyl-8-hydroxylquinoline (HMq) in the M-phenol mode. Cryst. Growth Des. 2010, 10, 4101–4108. 10.1021/cg100778w.

[ref35] McRobbie A.; Sarwar A. R.; Yeninas S.; Nowell H.; Baker M. L.; Allan D.; Luban M.; Muryn C. A.; Pritchard R. G.; Prozorov R.; Timco G. A.; Tuna F.; Whitehead G. F. S.; Winpenny R. E. P. Chromium chains as polydentate fluoride ligands for lanthanides. Chem. Commun. 2011, 47, 6251–6253. 10.1039/c1cc11516b. 21552574

[ref36] Kalmbach J.; Wang C.; You Y.; Forster C.; Schubert H.; Heinze K.; Resch-Genger U.; Seitz M. Near-IR to Near-IR Upconversion Luminescence in Molecular Chromium Ytterbium Salts. Angew. Chem., Int. Ed. 2020, 59, 18804–18808. 10.1002/anie.202007200. PMC7589230 32558117

[ref37] Doistau B.; Jimenez J. R.; Guerra S.; Besnard C.; Piguet C. Key Strategy for the Rational Incorporation of Long-Lived NIR Emissive Cr(III) Chromophores into Polymetallic Architectures. Inorg. Chem. 2020, 59, 1424–1435. 10.1021/acs.inorgchem.9b03163. 31909978

[ref38] Doistau B.; Jimenez J. R.; Daku L. M. L.; Piguet C. Complex-as-Ligand Strategy as a Tool for the Design of a Binuclear Nonsymmetrical Chromium(III) Assembly: Near-Infrared Double Emission and Intramolecular Energy Transfer. Inorg. Chem. 2022, 61, 11023–11031. 10.1021/acs.inorgchem.2c01940. 35820089

[ref39] Waki M.; Abe H.; Inouye M. Helix Formation in Synthetic Polymers by Hydrogen Bonding with Native Saccharides in Protic Media. Chem.—Eur. J. 2006, 12, 7839–7847. 10.1002/chem.200600315. 16847986

[ref40] Jäger M.; Kumar R. J.; Görls H.; Bergquist J.; Johansson O. Facile Synthesis of Bistridentate Ru^II^ Complexes Based on 2,6-Di(quinolin-8-yl)pyridyl Ligands: Sensitizers with Microsecond 3MLCT Excited State Lifetimes. Inorg. Chem. 2009, 48, 3228–3238. 10.1021/ic802342t. 19254036

[ref41] Norris M. R.; Concepcion J. J.; Glasson C. R. K.; Fang Z.; Lapides A. M.; Ashford D. L.; Templeton J. L.; Meyer T. J. Synthesis of Phosphonic Acid Derivatized Bipyridine Ligands and Their Ruthenium Complexes. Inorg. Chem. 2013, 52, 12492–12501. 10.1021/ic4014976. 24187928

[ref42] Ashford D. L.; Brennaman M. K.; Brown R. J.; Keinan S.; Concepcion J. J.; Papanikolas J. M.; Templeton J. L.; Meyer T. J. Varying the Electronic Structure of Surface-Bound Ruthenium(II) Polypyridyl Complexes. Inorg. Chem. 2015, 54, 460–469. 10.1021/ic501682k. 25532589

[ref43] Doistau B.; Collet G.; Acuna Bolomey E.; Sadat-Noorbakhsh V.; Besnard C.; Piguet C. Heteroleptic Ter-Bidentate Cr(III) Complexes as Tunable Optical Sensitizers. Inorg. Chem. 2018, 57, 14362–14373. 10.1021/acs.inorgchem.8b02530. 30376321

[ref44] Richardson C.; Reed C. A. Synthesis of meso-Extended Tetraarylporphyrins. J. Org. Chem. 2007, 72, 4750–4755. 10.1021/jo070191p. 17530895 PMC2543090

[ref45] Nettekoven M.; Jenny C. The Development of a Practical and Reliable Large-Scale Synthesis of 2,6-Diamino-4-bromopyridine. Org. Process Res. Dev. 2003, 7, 38–43. 10.1021/op020085j.

[ref46] Jimenez J.-J.; Doistau B.; Besnard C.; Piguet C. Versatile Heteroleptic Bis-Terdentate Cr(III) Chromophores Displaying Room Temperature Millisecond Excited State Lifetimes. Chem. Commun. 2018, 54, 13228–13231. 10.1039/C8CC07671E. 30406237

[ref47] Jiménez J.-R.; Poncet M.; Doistau B.; Besnard C.; Piguet C. Luminescent polypyridyl heteroleptic Cr^III^ complexes with high quantum yields and long excited state lifetimes. Dalton Trans. 2020, 49, 13528–13532. 10.1039/D0DT02872J. 32968750

[ref48] Barker K. D.; Barnett K. A.; Connell S. M.; Glaeser J. W.; Wallace A. J.; Wildsmith J.; Herbert B. J.; Wheeler J. F.; Kane-Maguire N. A. P. Synthesis and characterization of heteroleptic [Cr(diimine)_3_]^3+^ complexes. Inorg. Chim. Acta 2001, 316, 41–49. 10.1016/S0020-1693(01)00377-2.

[ref49] Jimenez J. R.; Doistau B.; Cruz C. M.; Besnard C.; Cuerva J. M.; Campana A. G.; Piguet C. Chiral Molecular Ruby [Cr(dqp)_2_]^3+^ with Long-Lived Circularly Polarized Luminescence″. J. Am. Chem. Soc. 2019, 141, 13244–13252. 10.1021/jacs.9b06524. 31353904

[ref50] Jiménez J.-R.; Poncet M.; Míguez-Lago S.; Grass S.; Lacour J.; Besnard C.; Cuerva J. M.; Campaña A. G.; Piguet C. Bright Long-Lived Circularly Polarized Luminescence in Chiral Chromium(III) Complexes. Angew. Chem., Int. Ed. 2021, 60, 10095–10102. 10.1002/anie.202101158. 33704880

[ref51] Sinha N.; Yaltseva P.; Wenger O. S. The Nephelauxetic Effect Becomes an Important Design Factor for Photoactive First-Row Transition Metal Complexes. Angew. Chem., Int. Ed. 2023, 62, e202303864 10.1002/anie.202303864. 37057372

[ref52] Wenger O. S. Photoactive Complexes with Earth-Abundant Metals. J. Am. Chem. Soc. 2018, 140, 13522–13533. 10.1021/jacs.8b08822. 30351136

[ref53] Benchohra A.; Chong J. L.; Cruz C. M.; Besnard C.; Guénée L.; Rosspeintner A.; Piguet C. Additional Insights into the Design of Cr(III) Phosphorescent Emitters Using 6-Membered Chelate Ring Bis(imidazolyl) Didentate Ligands. Inorg. Chem. 2024, 63, 3617–3629. 10.1021/acs.inorgchem.3c03422. 38206181

[ref54] Maeder M.; King P. Analysis of chemical processes, determination of the reaction mechanism and fitting of equilibrium and rate constants. In Chemometrics in Practical Applications; Varmuza K., Ed.; Intech, 2012; pp 41–62.

[ref55] Gampp H.; Maeder M.; Meyer C. J.; Zuberbuehler A. D. Calculation of equilibrium constants from multiwavelength spectroscopic data. III. Model-free analysis of spectrophotometric and ESR titrations. Talanta 1985, 32, 1133–1139. 10.1016/0039-9140(85)80238-1. 18963968

[ref56] Gampp H.; Maeder M.; Meyer C. J.; Zuberbuehler A. D. Calculation of equilibrium constants from multiwavelength spectroscopic data - IV. Model-free least-squares refinement by use of evolving factor analysis. Talanta 1986, 33, 943–951. 10.1016/0039-9140(86)80233-8. 18964236

[ref57] Clifford S.; Lawrance G. A.; Neuhold Y.-M.; Maeder M. Conjoint analysis of kinetic and equilibrium data for mechanistic elucidation in polynuclear complexation reactions, exemplified by metal(II) helicate complex formation. Aust. J. Chem. 2010, 63, 141–144. 10.1071/CH09316.

[ref58] Grenthe I. Stability Relationships among Rare Earth Dipicolinates. J. Am. Chem. Soc. 1961, 83, 360–364. 10.1021/ja01463a024.

[ref59] Piguet C. Microscopic Thermodynamic Descriptors for Rationalizing Lanthanide Complexation Processes. In Handbook on the Physics and Chemistry of Rare Earths; GschneidnerJr K. A.; Bünzli J.-C. G.; Pecharsky V. K., Eds.; Elsevier Science: Amsterdam, 2015; Vol. 47, pp 209–271.

[ref60] Alderighi L.; Gans P.; Ienco A.; Peters D.; Sabatini A.; Vacca A. Hyperquad simulation and speciation (HySS): a utility program for the investigation of equilibria involving soluble and partially soluble species. Coord. Chem. Rev. 1999, 184, 311–318. 10.1016/S0010-8545(98)00260-4.

[ref61] Strickler S. J.; Berg R. A. Relationship between Absorption Intensity and Fluorescence Lifetime of Molecules. J. Chem. Phys. 1962, 37, 814–822. 10.1063/1.1733166.

[ref62] Angulo G.; Grampp G.; Rosspeintner A. Recalling the appropriate representation of electronic spectra. Spectrochim. Acta Part a-Molecular and Biomolecular Spectroscopy 2006, 65, 727–731. 10.1016/j.saa.2006.01.007. 16495122

[ref63] McCusker J. K. Electronic structure in the transition metal block and its implications for light harvesting. Science 2019, 363, 484–488. 10.1126/science.aav9104. 30705184

[ref64] Hulliger F.; Landolt M.; Vetsch H. Rare-earth ferricyanides and chromicyanides LnT(CN)_6_·nH_2_O. J. Solid State Chem. 1976, 18 (3), 283–291. 10.1016/0022-4596(76)90107-9.

[ref65] Estrader M.; Ribas J.; Tangoulis V.; Solans X.; Font-Bardía M.; Maestro M.; Diaz C. Synthesis, Crystal Structure, and Magnetic Studies of One-Dimensional Cyano-Bridged Ln3+–Cr3+ Complexes with bpy as a Blocking Ligand. Inorg. Chem. 2006, 45 (20), 8239–8250. 10.1021/ic060942q. 16999423

[ref66] Andruh M.; Costes J.-P.; Diaz C.; Gao S. 3d-4f combined chemistry: synthetic strategies and magnetic properties. Inorg. Chem. 2009, 48, 3342–3359. 10.1021/ic801027q. 19361237

[ref67] Birk T.; Pedersen K. S.; Thuesen C. A.; Weyhermueller T.; Scha-Magnusen M.; Piligkos S.; Weihe H.; Mossin S.; Evangelisti M.; Bendix J. Fluoride bridges as structure-directing motifs in 3d-4f cluster chemistry. Inorg. Chem. 2012, 51, 5435–5443. 10.1021/ic300421x. 22497590

[ref68] Zare D.; Suffren Y.; Guénée L.; Eliseeva S. V.; Nozary H.; Aboshyan-Sorgho L.; Petoud S.; Hauser A.; Piguet C. Smaller than a nanoparticle with the design of discrete polynuclear molecular complexes displaying near-infrared to visible upconversion. Dalton Trans. 2015, 44, 2529–2540. 10.1039/C4DT02336F. 25357092

[ref69] Golesorkhi B.; Naseri S.; Guenee L.; Taarit I.; Alves F.; Nozary H.; Piguet C. Ligand-Sensitized Near-Infrared to Visible Linear Light Upconversion in a Discrete Molecular Erbium Complex. J. Am. Chem. Soc. 2021, 143 (37), 15326–15334. 10.1021/jacs.1c06865. 34498852

[ref70] Aboshyan-Sorgho L.; Cantuel M.; Petoud S.; Hauser A.; Piguet C. Optical sensitization and upconversion in discrete polynuclear chromium-lanthanide complexes. Coord. Chem. Rev. 2012, 256, 1644–1663. 10.1016/j.ccr.2011.12.013.

[ref71] Bolvin H.; Fürstenberg A.; Golesorkhi B.; Nozary H.; Taarit I.; Piguet C. Metal-Based Linear Light Upconversion Implemented in Molecular Complexes: Challenges and Perspectives. Acc. Chem. Res. 2022, 55 (3), 442–456. 10.1021/acs.accounts.1c00685. 35067044

