# Supplementary material for: Maximizing Nanoscale Downshifting Energy Transfer in a Metallosupramolecular Cr(III)–Er(III) Assembly
Source: Inorg Chem. 2024 Aug 20;63(39):18345–54. doi: 10.1021/acs.inorgchem.4c02397 (PMC11445728; doi:10.1021/acs.inorgchem.4c02397)
Supplement: Supplementary file 1 — ic4c02397_si_001.pdf [file ic4c02397_si_001.pdf]

SUPPORTING INFORMATION

Maximizing Nanoscale Downshifting Energy Transfer in a Metallosupramolecular Cr(III)-Er(III) Assembly

Maxime Poncet,† Céline Besnard,‡ Juan-Ramón Jiménez*§ and Claude Piguet*†

†Department of Inorganic and Analytical Chemistry, University of Geneva, 30 quai E. Ansermet, CH-1211 Geneva 4, Switzerland

‡Laboratory of Crystallography, University of Geneva, 24 quai E. Ansermet, CH-1211 Geneva 4, Switzerland.

§Departamento de Química inorgánica, Facultad de Ciencias, Universidad de Granada and Unidad de Excelencia en Química (UEQ), Avda. Fuente Nueva s/n, 18071, Granada, Spain

Supporting Information

(34 pages)

Appendix 1: Experimental section.

Crystallographic measurements.

Summary of crystal data, intensity measurements and structure refinements for ligand **10** and complex [Cr(**dqp**)(H_2-**L1**)](CF_3SO_3)$_3$·3.5H_2O are collected in Tables S1-S3, S5-S6 with pertinent bond lengths, bond angles. ORTEP views with pertinent numbering schemes are gathered in Figures S2-S3 and S5-S7. The crystals were mounted on Hampton cryoloops with protection oil. X-ray data collections were performed with a XtaLAB Synergy-S diffractometer equipped with a hybrid pixel array “hypix arc 150” detector. The structures were solved by using the dual-space methods in SHELXT. [A1-5]. Full-matrix least-square refinements on F^2 were performed using SHELXL[A1-6] within the Olex2 software [A1-7] CCDC 2361449-2361450 contain the supplementary crystallographic data for this paper. These data can be obtained free of charge from The Cambridge Crystallographic Data Centre via https://www.ccdc.cam.ac.uk/structures/.

Synthetic procedure for the preparation of 4-((2,6-di(quinolin-8-yl)pyridin-4-yl)ethynyl)pyridine-2,6-dicarboxylate (10).

Synthesis of 4-hydroxy-2,6-dibromopyridine (2).

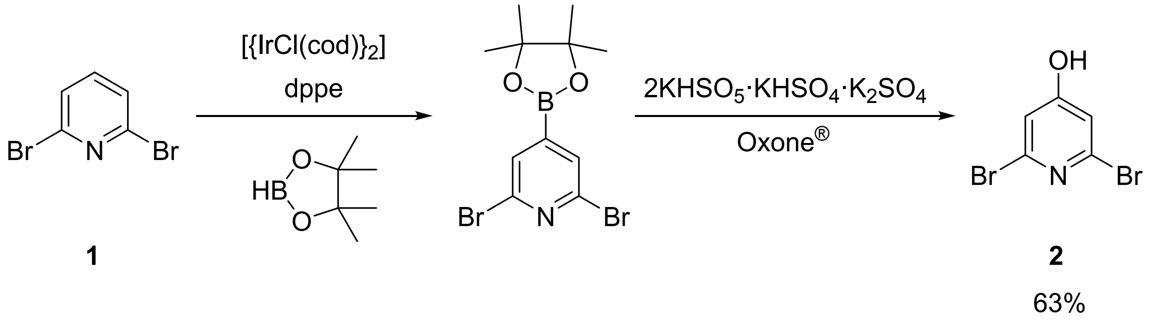

Pinacolborane (14.1 mL, 12 mmol, 5 eq.) was added under N_2 into a preloaded Schlenk containing [{IrCl(cod)}$_2$] (341.4 mg, 0.51 mmol, 0.02 eq.), 1,2-Bis(diphenylphosphino)ethane DPPE (408.7 mg, 1.03 mmol, 0.04 eq.) and 2,6-dibromopyridine **1** (6.00 g, 25.5 mmol). The resulting mixture was heated at 130°C for 5h under vigorous stirring. After solvent evaporation, the residue was diluted in THF (95 mL) and aqueous Oxone® (17g, 28.05 mmol, 1.1 eq.) was slowly added. After 10 min of stirring at RT, the mixture was neutralised with aqueous $NaHSO_3$. The water phase was extracted with Et_2O (3x250mL) and the combined organic phases were dried over Na_2SO_4. The solvent was removed under vacuum and the crude product was purified by silica column chromatography (AcOEt:cyclohexane, 1:5) followed by a wash with cold cyclohexane (2x5 mL) yielding 4-hydroxy-2,6-dibromopyridine **2** as a white powder (4.01 g, 16.04 mmol, 62.9%).

^{1}H NMR (MeCN-d$_3$, 400 MHz): δ (ppm); 7.04 (s, 2H); 8.63 (s, 1H). ESI-MS (CH_3CN) m/z: [**2**+H]$^+$ calc: 253.9, found: 254.1.

Synthesis of 2,6-dibromo-4-methoxypyridine (3).

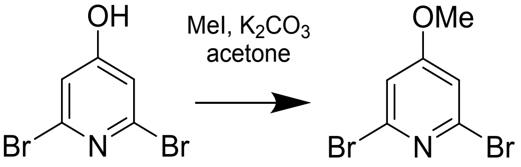

2 **3**

98%

4-hydroxy-2,6-dibromopyridine **2** (6.65 g, 26.30 mmol) and K_2CO_3 (29.1 g, 210.5 mmol, 8 eq.) were loaded into a flask containing 60 mL of acetone. Under stirring, methyl iodide (1.8 mL, 28.92 mmol, 1.1 eq.) was added dropwise, and the mixture refluxed for 16h. After filtration and washing of the remaining solid with acetone, the solution was evaporated to dryness. Dichloromethane was added and after filtration to remove the remaining excess of K_2CO_3, the solvent was removed under vacuum, yielding 2,6-dibromo-4-methoxypyridine **3** (6.89 g, 25.84 mmol, 98.3%) as a white powder.

^{1}H NMR (MeCN-d$_3$, 400 MHz): δ (ppm); 3.87 (s, 3H); 7.18 (s, 2H).

ESI-MS (CH_3CN) m/z: [**3**+H]$^+$ calc: 267.9, found: 268.0.

Synthesis of 8,8'-(4-methoxypyridine-2,6-diyl)diquinoline (4).

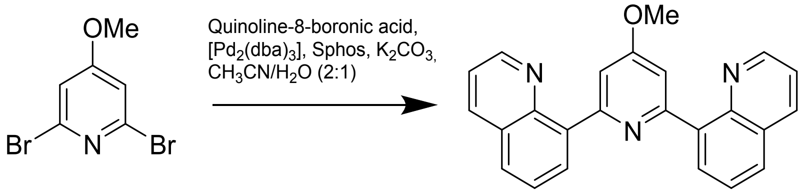

3 **4**

76%

2,6-Dibromo-4-methoxypyridine **3** (653.9 mg, 2.45 mmol), quinoline-8-boronic acid (899.6 mg, 5.20 mmol, 2.1 eq.), [$Pd_2(dba)_3$] (dba = dibenzylideneacetone) (116 mg, 0.13 mmol, 0.05 eq.), 2-dicyclohexylphosphino-2',6'-dimethoxybiphenyl (SPhos) (164.6 mg, 0.40 mmol, 0.15 eq.), K_2CO_3 (2085.8 mg, 15.09 mmol, 6.16 eq.) in a mixture of acetonitrile:water (15 mL 2:1) were loaded into a microwave vial. The mixture was bubble with N_2 for 30 min and heated to 140°C for 2h in a microwave. After cooling, ethyl acetate was added to the solution of acetonitrile:water, which was washed with water (3x300 mL). The organic phase was dried over Na_2SO_4, filtered and the solvent was removed under vacuum. The crude product was washed with a minimum volume of cold acetonitrile to remove impurities (2x5 mL) yielding 4-methoxy-2,6-di(quinoline-8-yl)pyridine **4** (680.0 mg, 1.87 mmol, 76.3%) as a white powder. ^{1}H NMR (MeCN-d$_3$, 400 MHz): δ (ppm); 4.00 (s, 3H); 7.57 (dd, 2H); 7.73 (dd, 2H); 7.76 (s, 2H); 8.03 (dd, 2H); 8.24 (dd, 2H); 8.40 (dd, 2H); 8.99 (dd, 2H). ESI-MS (CH_3CN) m/z: [**4**+H]$^+$ calc: 364.4, found: 363.6.

Synthesis of 8,8'-(4-bromopyridine-2,6-diyl)diquinoline (5).

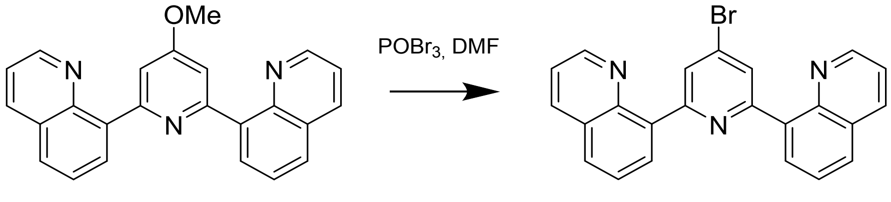

4 **5**

84%

A solution of anhydrous DMF (20 mL) containing 8,8'-(4-methoxypyridine-2,6-diyl)diquinoline **4** (1.04g, 2.86 mmol) was added dropwise to a cold solution (0°C) of $POBr_3$ (2.10g, 7.32 mmol, 2.56 eq.) in anhydrous DMF (40 mL). After 10 min at this temperature, the mixture was heated up to 105°C for 6h under an inert atmosphere. The reaction mixture was cooled to 0°C and water (50 mL) were slowly added. The solution was neutralised with $NaHCO_3$ and the formed solid was filtered on membrane. The resulting solid was washed by flowing acetone on the precipitate and the filtrate was evaporated and purified by column chromatography on silica (2% MeOH in dichloromethane) yielding 8,8'-(4-bromopyridine-2,6-diyl)diquinoline **5** (1.00 g, 2.41 mmol, 84%) as an off-white powder.

^{1}H NMR (MeCN-d_3, 400 MHz): δ (ppm); 7.59 (s, 3H); 7.76 (dd, 2H); 8.08 (dd, 2H); 8.32 (dd, 2H); 8.43 (dd, 2H); 8.46 (s, 2H); 9.01 (dd, 2H).

ESI-MS (CH_3CN) m/z: [**5**+H]$^+$ calc: 413.2, found: 412.3.

Synthesis of 8,8'-(4-ethynylpyridine-2,6-diyl)diquinoline (6)

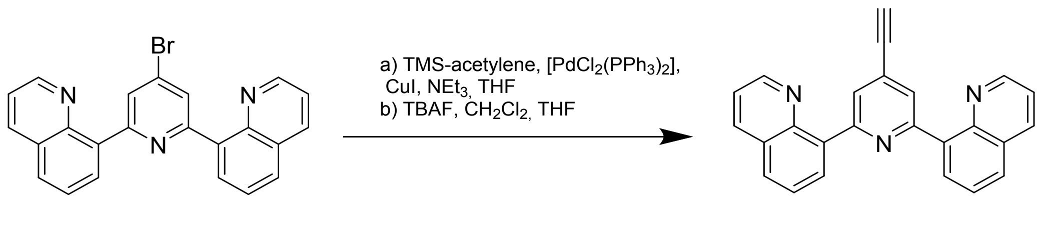

5 **6**

69%

A solution of degassed triethylamine (10 mL, 71.75 mmol) and dried THF (20 mL) was transferred under N_2 into a preloaded Schlenk containing 8,8'-(4-bromopyridine-2,6-diyl)diquinoline **5** (400 mg, 0.97 mol), $PdCl_2(PPh_3)_2$ (60 mg, 0.09 mmol, 0.08 eq.) and CuI (30 mg, 0.16 mmol, 0.17 eq.). TMS-acetylene (0.2 mL, 1.44 mmol, 1.5 eq.) were added and the reaction was heated at 75°C for 16h. After solvent evaporation, the crude residue was dissolved in dichloromethane and tris(2-aminoethyl)amine (0.3 mL) was added to complex the copper in solution. The organic phase was extracted with water (3 x 250 mL), dried over Na_2SO_4, filtered and the solvent removed under vacuum. A solution of tetra-n-butylammonium fluoride (TBAF) 1M in THF was added (1.5 mL, 1.5 mmol, 1.5 eq.), left stirring at room temperature for 1h and extracted with water (4 x 250 mL). The organic phase was dried over

Na_2SO_4. Methanol (5 mL) was added to the crude product for dissolving the impurities and the solid was filtered out yielding 8,8'-(4-ethynylpyridine-2,6-diyl)diquinoline **6** as an off-white powder (236.9 mg, 69%).

^{1}H NMR ($CDCl_3$, 400 MHz): δ (ppm); 3.30 (s, 1H); 7.49 (dd, 2H); 7.69 (dd, 2H); 7.92 (dd, 2H); 8.25 (s, 2H); 8.27 (dd, 2H); 9.05 (dd, 2H).

ESI-MS (CH_3CN) m/z: [**6**+H]$^+$ calc: 358.4, found: 357.8.

Synthesis of diethyl 4-bromopyridine-2,6-dicarboxylate (8).

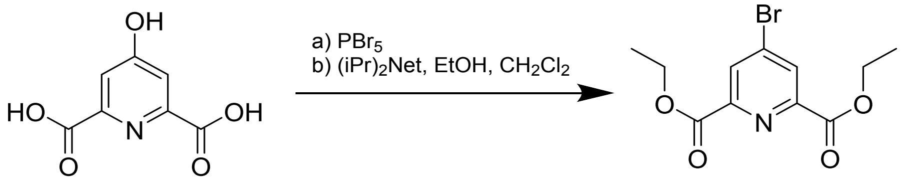

7 **8**

62%

In a Schlenk tube, chelidamic acid **7** (3.60 g, 19.66 mmol) and PBr_5 (24.82 g, 57.66 mmol, 3.02 eq.) were heated at 80°C for 4h under N_2 atmosphere. After cooling, distilled DCM (40 mL) was added to the resulting mixture and the whole was cooled in an ice bath. Once cooled, the solution was filtered using a Schlenk techniques under N_2 and directly added to a cold solution (0°C) containing ethanol (20 mL), (*i*-Pr)$_2$NEt (20 mL) and distilled dichloromethane (20 mL). The mixture was stirred at 50°C for 16h. After solvent evaporation, the crude residue was dissolved in dichloromethane, extracted with water (5x500 mL) and the organic phase dried over Na_2SO_4. The product was purified by column chromatography on silica (EtOAc:cyclohexane 40/60) yielding diethyl 4-bromopyridine-2,6-dicarboxylate **8** (3.66 g, 12.14 mmol, 62%) as a white powder.

^{1}H NMR ($CDCl_3$, 400 MHz): δ (ppm); 1.48 (t, 6H); 4.52 (dd, 4H); 8.54 (s, 2H).

ESI-MS (CH_3CN) m/z: [**8**+H]$^+$ calc: 303.1, found: 301.9.

Synthesis of diethyl 4-ethynylpyridine-2,6-dicarboxylate (9)

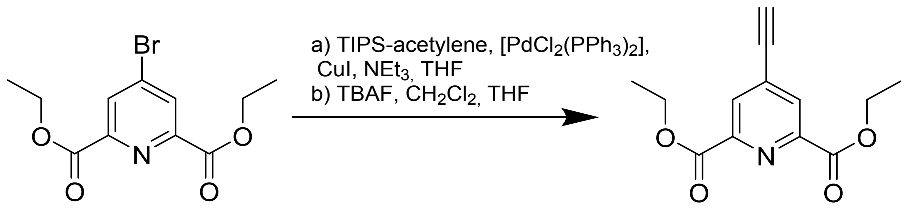

8 **9**

65%

Triethylamine (40 mL) and DMSO (40 mL) were bubbled with Argon for 10 min. The degassed solution was then transferred via canula into an inert preloaded Schlenk tube containing diethyl 4-bromopyridine-2,6-dicarboxylate **8** (2.90 g, 9.60 mmol), $Pd(PPh_3)_4$ (348.9 mg, 0.302 mmol, 0.03 eq.), sodium L-ascorbate (119 mg, 0.60 mmol, 0.06 eq.) and $CuSO_4 \cdot 5H_2O$ (25.6 mg, 0.10 mmol, 0.03 eq.).

To the resulting solution was added Si(^{i}Pr)$_3$-acetylene (2.89 mL, 12.48 mmol, 1.3 eq.) and the mixture was heated at 100°C for 3h. After evaporation of triethylamine, the solution was dissolved in dichloromethane and tris(2-aminoethyl)amine (1 mL, 6.68 mmol) was added. The solution was extracted with water (3x1000 mL), dried over Na_2SO_4 and evaporated to dryness. Dichloromethane (100 mL) was added to solubilise the crude. To this concentrated solution was added TBAF 1M in THF (18.7 mL, 18.7 mmol, 1.5 eq.) and stirred at RT for 20 min. After extraction with water (2x1000 mL), the organic phase was dried over Na_2SO_4, filtered and evaporated under vacuum to dryness. The crude product was purified by filtration after being solubilised in cyclohexane. Column chromatography on silica (0.5% MeOH in dichloromethane) yields diethyl 4-ethynylpyridine-2,6-dicarboxylate **9** (1553 mg, 6.29 mmol, 65%) as a white powder.

^{1}H NMR ($CDCl_3$, 400 MHz): δ (ppm); 1.48 (t, 6H); 3.49 (s, 1H); 4.50 (dd, 4H); 8.32 (s, 2H).

ESI-MS (CH_3CN) m/z: [**9**+H]$^+$ calc: 248.2, found: 247.9. Elemental analysis: Calcd for $C_{13}H_{13}NO_4$: C, 63.15; H, 5.30; N, 5.67. Found C, 63.18; H, 5.25; N, 5.54.

Synthesis of diethyl 4-((2,6-di(quinolin-8-yl)pyridin-4-yl)ethynyl)pyridine-2,6-dicarboxylate (10).

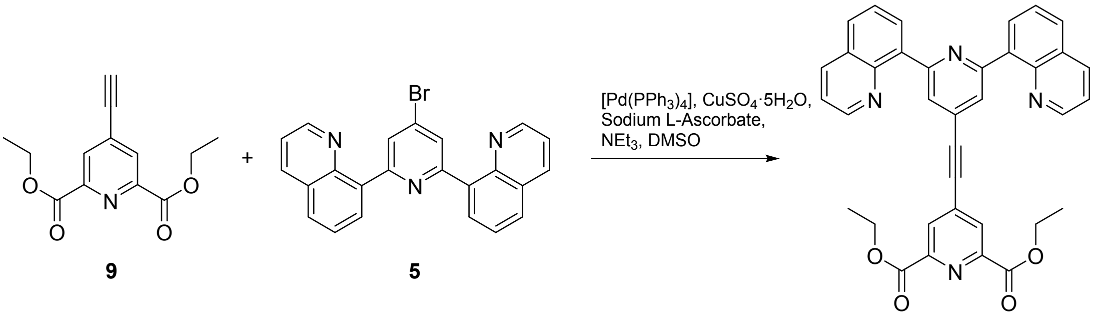

9 5 10

78%

A mixture of NEt_3 and DMSO (1:1, 20mL total per vial, 3 vials in total) was put into the microwave vial and was bubbled with N_2 for 10 min. **5** (896 mg, 2.17 mmol), $CuSO_4 \cdot 5H_2O$ (6.8 mg, 0.03 mmol), sodium L-ascorbate (27.7 mg, 0.14 mmol) were then added to the solution and the solution was bubbled again for 5 min. **9** (578 mg, 2.34 mmol) and $Pd(PPh_3)_4$ (76.9 mg, 0.067 mmol) were added to the mixture. The vial was sealed and put into the microwave at 120°C for 10 min. The NEt_3 was evaporated and the solid was dissolved in DCM and tris(2-aminoethyl)amine was added to complex the copper in solution (0.1 mL). The DCM phase was washed with water (3x600 mL). The combined organic phases were dried over Na_2SO_4, filtered and evaporated. A column on silica was prepared (eluent: 35% Ethyl acetate, 35% Cyclohexane, 30% DCM). Along with the product, a yellow impurity eluted that could be eliminated by being solubilised in ethyl acetate (980 mg, 1.69 mmol, 78%). Single crystals suitable for X-ray diffraction were obtained by slow evaporation of EtOAc.

^{1}H NMR (MeCN-d$_3$, 400 MHz): δ (ppm); 1.40 (t, 6H), 4.44 (dd, 4H); 7.58 (dd, 2H); 7.75 (dd, 2H); 8.06 (dd, 2H); 8.31 (dd, 2H); 8.41 (m, 6H); 9.00 (dd, 2H).
ESI-MS (CH$_3$CN) m/z: [**10**+H]$^+$ calc: 579.6, found: 579.3. Elemental analysis: Calcd for $C_{36}H_{26}N_4O_4$: C, 74.73; H, 4.53; N, 9.68. Found C, 74.21; H, 4.41; N, 9.32.

Synthesis of the complex-as-ligand

Synthesis of 2,6-di(quinoline-8-yl)pyridine (dqp).

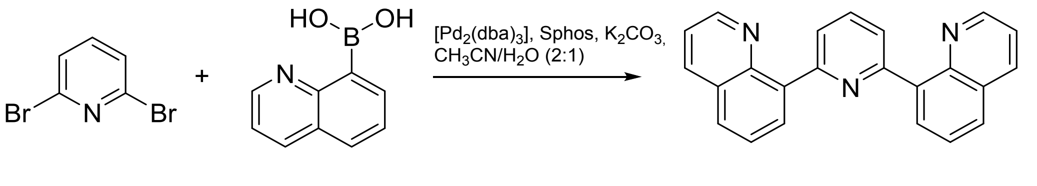

2,6-dibromopyridine (731.5 mg, 3.13 mmol, 1 eq), quinoline-8-boronic acid (1178.1 mg, 6.81 mmol, 2.2 eq), [Pd(dba)$_2$] (122.8 mg, 0.21 mmol, 7 mol%), Sphos (171.2 mg, 0.417 mmol, 13.3 mol%) and Potassium carbonate (2048 mg, 14.82 mmol, 4.74 eq) were weighed and split into two different 20 mL MW vial. 10 mL ACN and 5 mL H_2O were added. The solutions were each put into the MW for 2h at 140°C. The resulting combined mixture were extracted with water (3x250 mL) and EtOAc. The organic fraction was dried over Na_2SO_4 and evaporated to dryness. Cold EtOAc was used to solubilise the impurity. Filtration led to the isolation of 2,6-di(quinoline-8-yl)pyridine (**dqp**: 819 mg, 2.46 mmol, 79%) as an off-white powder.
^{1}H NMR (CDCl$_3$, 400 MHz): δ (ppm); 7.44 (q, 2H); 7.66 (dd, 2H); 7.88 (dd, 2H); 7.94 (dd, 2H); 8.11 (s, 2H); 8.13 (s, 2H); 8.23 (dd, 2H), 8.27 (dd, 2H); 9.00 (dd, 2H).
ESI-MS (CH$_3$CN) m/z: [dqp+H]$^+$ calc: 334.1, found: 334.1. Elemental analysis: Calcd for $C_{23}H_{15}N_3$: C, 82.86; H, 4.54; N, 12.60. Found C, 82.58; H, 4.50; N, 12.55.

Synthesis of $Cr(CF_3SO_3)_2 \cdot 2H_2O$.

$$Cr^0 + 2\,HSO_3CF_3 \xrightarrow{60°C,\ 24h,\ N_2,\ H_2O} Cr(SO_3CF_3)_2 \cdot 2H_2O$$

A solution of 80 mmol of trifluoromethanesulfonic acid (7 cm^3) in degassed water (50 cm^3) at 0 °C was added via a cannula onto electrolytic chromium (2 g, 38 mmol) under nitrogen. After stirring for 24 h at 60 °C, the blue solution was filtered and evaporated to dryness. The resulting blue powder was dried under vacuum (10^{-2}Torr/48 h) and transferred into a glove box. Spectrophotometric analysis of the chromium content after oxidation into chromate (fusion with KNO_3) gives %Cr = 13.41 corresponding to $[Cr(CF_3SO_3)_2]\cdot 2H_2O$ (%Cr = 13.47). Elemental analysis: Calcd for $[Cr(CF_3SO_3)_2]$: C, 6.22; H, 1.04; N, 0.00. Found C, 6.79; H, 0.64; N, 0.00.

Synthesis of [Cr(10)Cl$_3$].

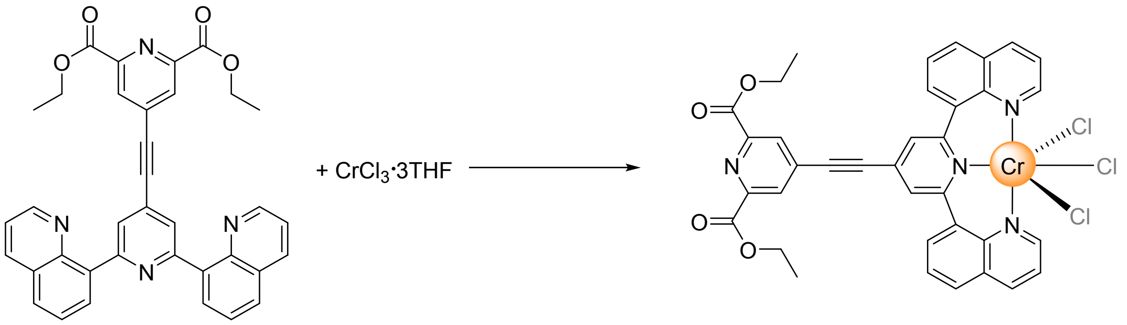

97%

Ligand **10** (490 mg, 0.85 mmol) was added into a solution of CrCl$_3$·3THF (320 mg, 0.85 mmol) in isopropanol (15 mL). The resulting purple solution was heated in a microwave at 160°C during 6 h. The mixture was filtered and the green solid was washed with warm ethanol (2 x 10 mL), dichloromethane (1x10 mL) and diethyl ether (2 x 20 mL). The product was dried under vacuum to obtain a green powder of [Cr(**10**)Cl$_3$] (603 mg, 0.82 mmol, 97%).

ESI-MS (DMF/CH$_3$CN) m/z: [Cr(**10**)Cl$_2$]$^+$ calc: 700.07, found: 700.1.

Elemental analysis: Calcd for: $C_{36}H_{26}Cl_3CrN_4O_4 \cdot 2H_2O$: C, 56.60; H, 3.50; N, 7.23. Found C, 55.94; H, 3.91; N, 7.25.

Synthesis of [Cr(10)(SO$_3$CF$_3$)$_3$].

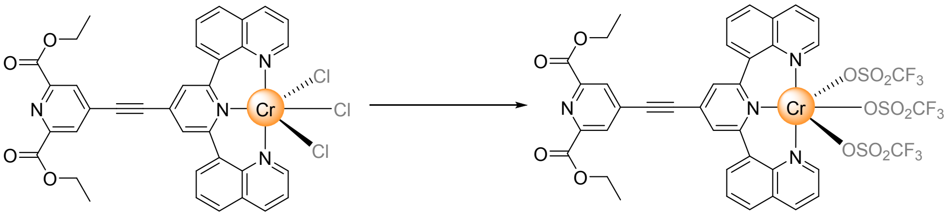

A mixture of [Cr(**10**)Cl$_3$] (107 mg, 0.14 mmol) and AgSO$_3$CF$_3$ (110.7 mg, 0.43 mmol) in distilled acetonitrile (1.5 mL) was heated under N$_2$ under microwave irradiation in a sealed cap at 140°C during 30 min. After cooling to room temperature, the resulting red solution was centrifuged and filtered to remove the AgCl generated during the reaction. ESI-MS (CH$_3$CN) *m/z*: [Cr(**10**)(SO$_3$CF$_3$)$_2$]$^+$·CH$_3$CN calc: 969.07, found: 969.0. This solution was used directly for the next step.

Synthesis of [Cr(dqp)(10)](SO$_3$CF$_3$)$_3$.

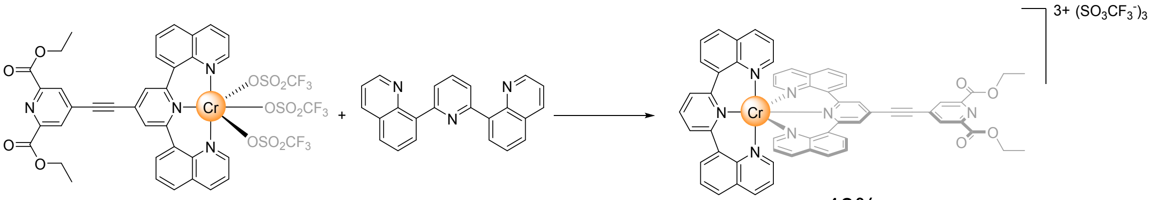

48%

A red solution of [Cr(**10**)(SO$_3$CF$_3$)$_3$] in distilled acetonitrile was loaded into a 5 ml microwave vial under N$_2$ containing 1 equivalent of the **dqp** ligand. The solution was heated under microwave

irradiation for 12h at 115°C. After cooling to room temperature, the grey precipitate was filtered off and rinsed with 1 mL of acetonitrile. Silica gel chromatography was done to isolate the product starting with ACN:H_2O (9:1). After elution of the neutral species, to the solvent mixture, 1 mL of concentrated $NaSO_3CF_3$ in H_2O was added for each 250mL of eluent. To accelerate the elution, 3 mL of concentrated $NaSO_3CF_3$ in H_2O was added for each 250mL of eluent. The complex was isolated by being solubilised in methanol (5 mL) and precipitated with the smallest amount of diethyl ether. The precipitate was filtered yielding [Cr(**dqp**)(**10**)]$(SO_3CF_3)_3$ as a bright orange powder (96 mg, 0.07 mmol, 48%).

Elemental Analysis: Calcd for $C_{62}H_{41}CrF_9N_7O_{13}S_3$·0.35 $NaSO_3CF_3$: C, 50.90; H, 2.81; N, 6.66 found C, 50.85; H, 3.04; N, 6.62.

ESI-MS (CH_3CN) *m/z*: [Cr(**dqp**)(**10**)]$(SO_3CF_3)_2^+$ calc: 1261.17, found: 1262.3; *m/z*: [Cr(**dqp**)(**10**)]$(SO_3CF_3)^{2+}$ calc: 556.1, found: 556.3.

Slow evaporation of a concentrated solution in methanol led to the formation of crystals of the trans-esterified complex suitable for XRD.

Synthesis of [Cr(dqp)(H_2-L1)]$(SO_3CF_3)_3$.

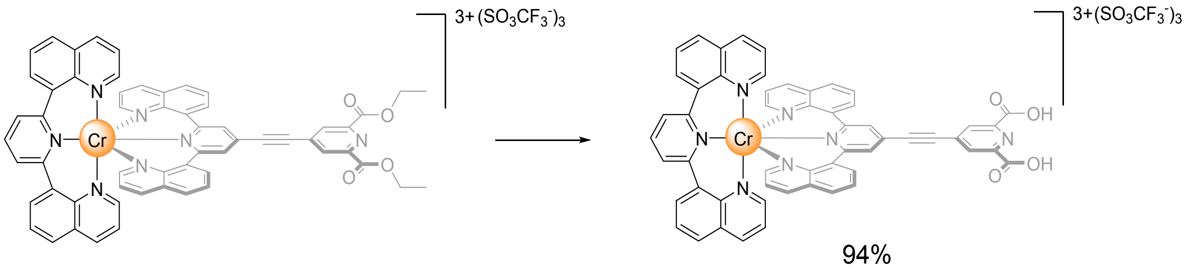

[Cr(**dqp**)(**10**)]$(SO_3CF_3)_3$ [Cr(**dqp**)(H_2-**L1**)]$(SO_3CF_3)_3$

To a solution of [Cr(**dqp**)(**10**)]$(SO_3CF_3)_3$ (92 mg, 0.065 mmol) in water (15 mL) was added NaOH (10.44 mg, 0.261 mmol). After agitating for 30 min at room temperature, HSO_3CF_3 (40.1 mg, 0.268 mmol) was added and the solvent was evaporated. The target complex was dissolved in methanol (5 mL) and precipitated with the smallest amount of diethyl ether. The precipitate was filtered yielding [Cr(**dqp**)(H_2-**L1**)]$(SO_3CF_3)_3$ as a bright orange powder (82.8 mg, 0.061 mmol, 94%).

Elemental Analysis: Calcd for 78% $C_{58}H_{33}CrF_9N_7O_{13}S_3$·1.35$H_2O$ ([Cr(**dqp**)(H_2-**L1**)]$(SO_3CF_3)_3$) and 22% $C_{57}H_{32}CrF_6N_7O_{10}S_2$·1.35 H_2O ([Cr(**dqp**)(H-**L1**)]$(SO_3CF_3)_2$): C, 52.23; H, 2.69; N, 7.40 found C, 52.17; H, 2.77; N, 7.49.

ESI-MS (CH_3CN) *m/z*: [Cr(**dqp**)(H_2-**L1**)]$(SO_3CF_3)_2^+$ calc: 1205.104, found: 1205.109.

Synthesis of the assemblies

Synthesis of [(dqpCrL1)$_3$Er](SO$_3$CF$_3$)$_6$.

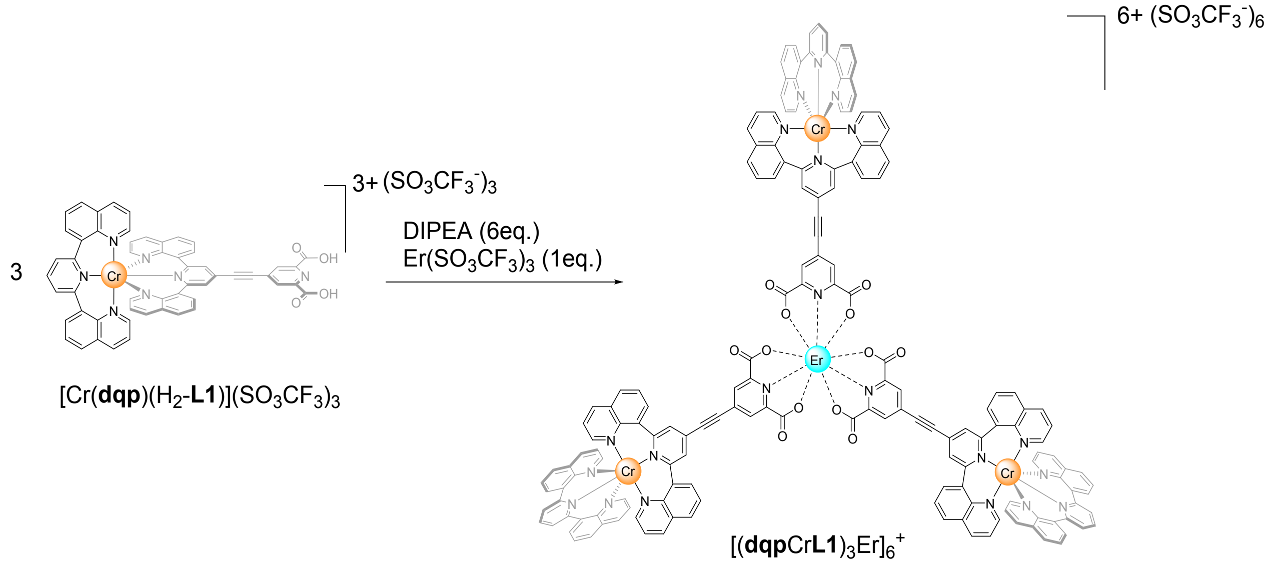

To a solution of [Cr(**dqp**)(H$_2$-**L1**)](SO$_3$CF$_3$)$_3$ (60.43 mg, 0.045 mmol, 3 eq.) in CH$_3$CN (2.8 mL) was added diisopropylethylamine DIPEA (11.6 mg, 0.9 mmol, 6 eq.) and Er(SO$_3$CF$_3$)$_3$ (9.22 mg, 0.015 mmol, 1eq.). After stirring for 5 min at room temperature, the target complex was precipitated with the smallest amount of diethyl ether. The precipitate was filtered yielding [(**dqp**Cr**L1**)$_3$Er](SO$_3$CF$_3$)$_6$ as a bright orange powder (63.9 mg, 0.041 mmol, 91.6%).

Elemental Analysis: Calcd for C$_{171}$H$_{93}$Cr$_3$ErF$_{18}$N$_{21}$O$_{30}$S$_6$ ([(**dqp**Cr**L1**)$_3$Er](SO$_3$CF$_3$)$_6$) + 2 C$_9$H$_{20}$F$_3$NO$_3$S (H-DIPEA(SO$_3$CF$_3$)) + 1.5 NaSO$_3$CF$_3$: C, 49.78; H, 2.92; N, 7.01. Found: C, 49.35 H, 3.04; N, 7.00.

Synthesis of [(dqpCrL1)$_3$Y](SO$_3$CF$_3$)$_6$:

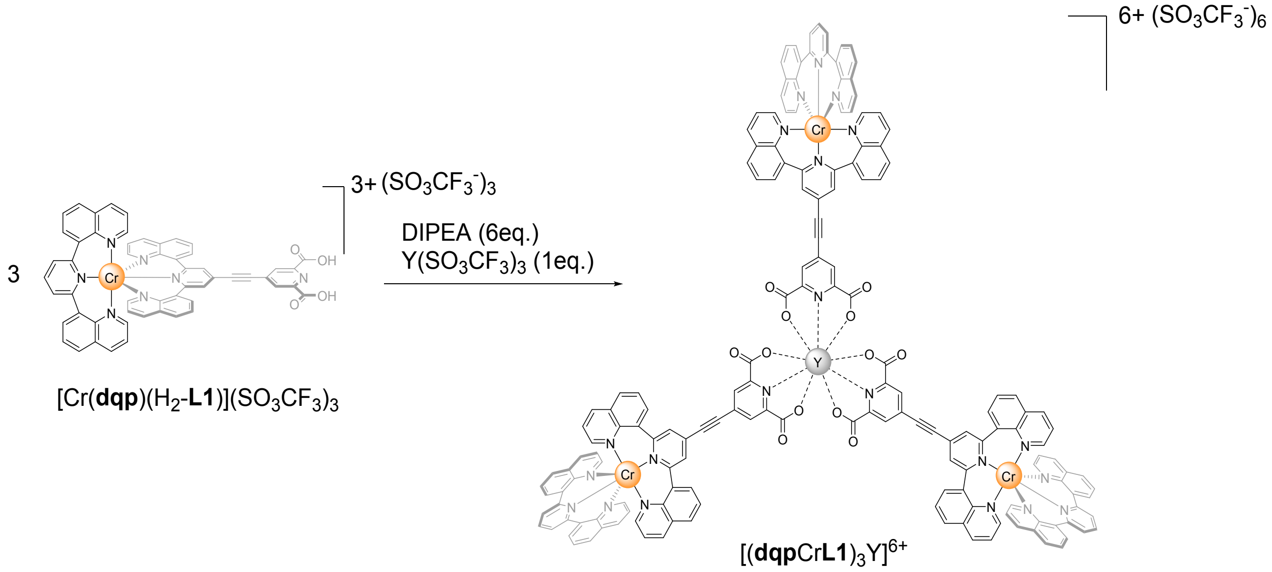

To a solution of [Cr(**dqp**)(H$_2$-**L1**)](SO$_3$CF$_3$)$_3$ (61.08 mg, 0.045 mmol, 3 eq.) in CH$_3$CN (2.8 mL) was added diisopropylethylamine DIPEA (11.6 mg, 0.9 mmol, 6 eq.) and Y(SO$_3$CF$_3$)$_3$ (8.16 mg, 0.015

mmol, 1eq.). After stirring for 5 min at room temperature, the wanted complex was precipitated with the smallest amount of diethyl ether. The precipitate was filtered yielding [(**dqp**Cr**L1**)$_3$Y](SO_3CF_3)$_6$ as a bright orange powder (63.6 mg, 0.041 mmol, 91.9%).

Elemental Analysis: Calcd for $C_{171}H_{93}Cr_3YF_{18}N_{21}O_{30}S_6$ ([(**dqp**Cr**L1**)$_3$Y](SO_3CF_3)$_6$) + 2 $C_9H_{20}F_3NO_3S$ (H-DIPEA(SO_3CF_3)) + 5 $NaSO_3CF_3$: C, 45.51; H, 2.62; N, 6.29. Found: C, 45.49, H, 2.65; N, 6.33.

References

(A1-1) Maeder, M.; King, P., Analysis of chemical processes, determination of the reaction mechanism and fitting of equilibrium and rate constants. In *Chemometrics in Practical Applications*, Varmuza, K., Ed. INTECH: 2012; pp 41-62.

(A1-2) Gampp, H.; Maeder, M.; Meyer, C. J.; Zuberbuehler, A. D., Calculation of equilibrium constants from multiwavelength spectroscopic data. III. Model-free analysis of spectrophotometric and ESR titrations. *Talanta* **1985**, *32*, 1133-1139.

(A1-3) Gampp, H.; Maeder, M.; Meyer, C. J.; Zuberbuehler, A. D., Calculation of equilibrium constants from multiwavelength spectroscopic data - IV. Model-free least-squares refinement by use of evolving factor analysis. *Talanta* **1986**, *33*, 943-951.

(A1-4) Clifford, S.; Lawrance, G. A.; Neuhold, Y.-M.; Maeder, M., Conjoint analysis of kinetic and equilibrium data for mechanistic elucidation in polynuclear complexation reactions, exemplified by metal(II) helicate complex formation. *Aust. J. Chem.* **2010**, *63*, 141-144.

(A1-5) Sheldrick, G. M., *SHELXT* – Integrated Space-Group and Crystal-Structure Determination. *Acta Cryst. A* **2015**, *64*, 3-8.

(A1-6) Sheldrick, G. M., Crystal structure refinement with SHELXL. *Acta Cryst. C* **2015**, *71*, 3-8.

(A1-7) Dolomanov, O.V.; Bourhis, L. J.; Gildea, R. J.; Howard, J. A. K.; Puschmann, H., OLEX2: A Complete Structure Solution, Refinement and Analysis Program. *J. Appl. Cryst.* **2009**, *42*, 339-341.

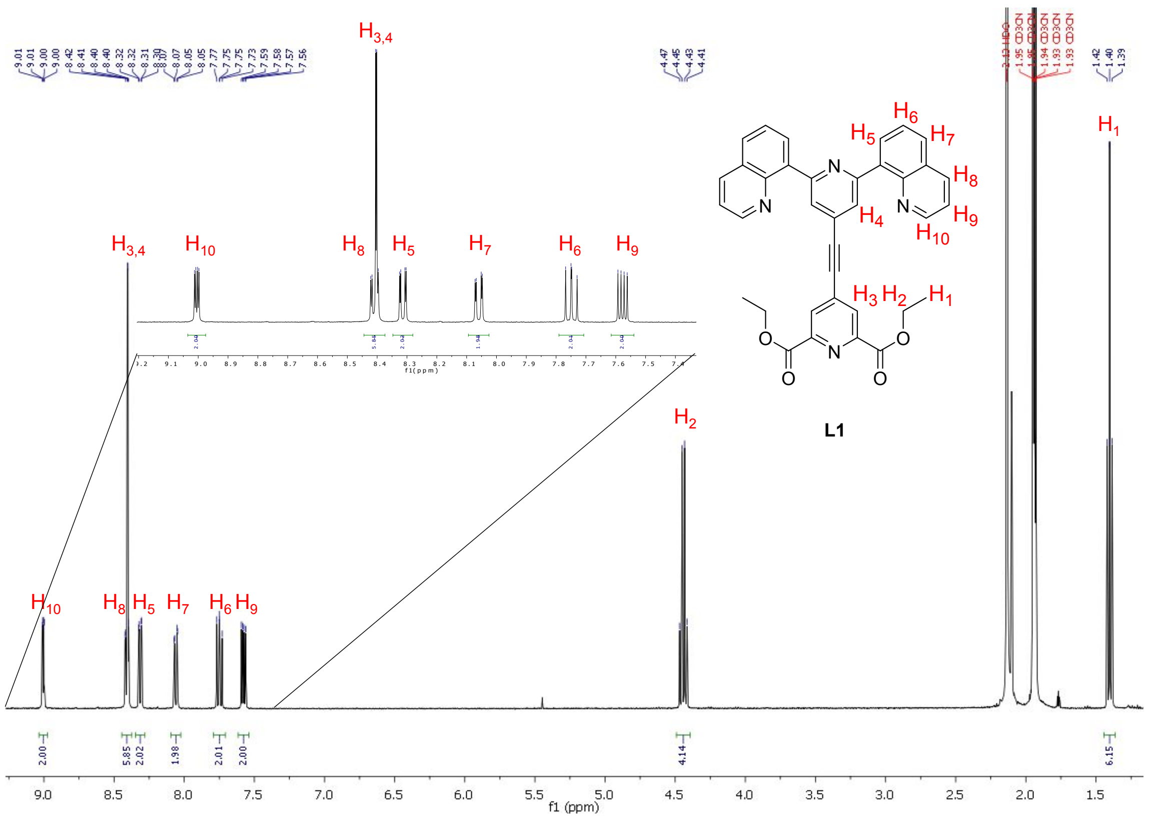

Figure S1. ^{1}H NMR of **10** (MeCN-d_3, 298K, 400 MHz)

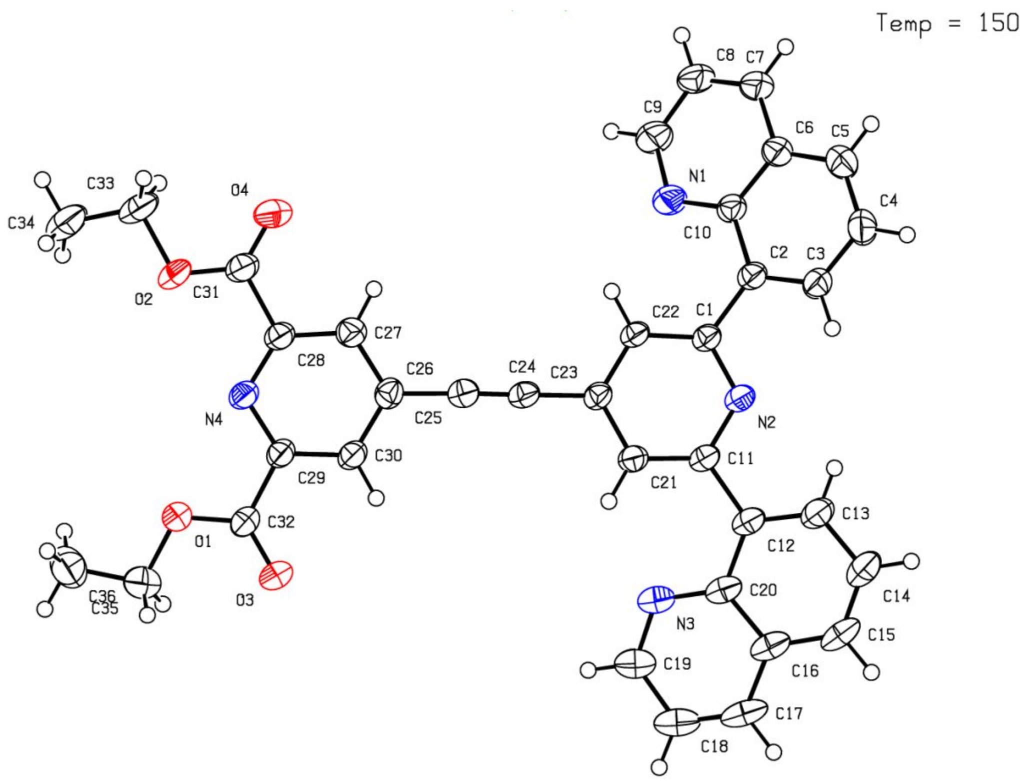

Figure S2. Crystal structure of the organic ligand **10**. Displacement ellipsoids are shown at 50 percent probability level.

Table S1. Crystal data and structure refinement for **10**.

**CCDC number**	**2361450**
Empirical formula	$C_{36}H_{26}N_4O_4$
Formula weight	578.61
Temperature/K	150.00(10)
Crystal system	monoclinic
Space group	$P2_1/n$
a/Å	10.9057(2)
b/Å	21.9232(4)
c/Å	11.9716(3)
α/°	90
β/°	98.973(2)
γ/°	90
Volume/Å^3	2827.24(10)
Z	4
ρ_{calc}g/cm^3	1.359
μ/mm^{-1}	0.730
F(000)	1208.0
Crystal size/mm^3	0.239 × 0.197 × 0.039
Radiation	Cu Kα (λ = 1.54184)
2Θ range for data collection/°	8.066 to 148.174
Index ranges	$-13 \leq h \leq 11$, $-27 \leq k \leq 26$, $-14 \leq l \leq 14$
Reflections collected	34481
Independent reflections	5691 [R_{int} = 0.0353, R_{sigma} = 0.0171]
Data/restraints/parameters	5691/0/400
Goodness-of-fit on F^2	1.049
Final R indexes [I>=2σ (I)]	R_1 = 0.0641, wR_2 = 0.1704
Final R indexes [all data]	R_1 = 0.0693, wR_2 = 0.1741
Largest diff. peak/hole / e Å^{-3}	0.80/-0.34

Table S2. Bond Lengths for **10**.

Atom	Atom	Length/Å	Atom	Atom	Length/Å
O1	C32	1.330(3)	C11	C12	1.496(3)
O1	C35	1.473(3)	C11	C21	1.391(3)
O2	C31	1.319(3)	C12	C13	1.374(3)
O2	C33	1.476(3)	C12	C20	1.432(3)
O3	C32	1.204(3)	C13	C14	1.407(3)
O4	C31	1.203(3)	C14	C15	1.358(4)
N1	C9	1.318(3)	C15	C16	1.410(4)
N1	C10	1.364(3)	C16	C17	1.413(4)
N2	C1	1.338(2)	C16	C20	1.422(3)
N2	C11	1.347(3)	C17	C18	1.352(4)
N3	C19	1.318(3)	C18	C19	1.405(3)
N3	C20	1.369(3)	C21	C23	1.396(3)
N4	C28	1.338(3)	C22	C23	1.393(3)
N4	C29	1.338(3)	C23	C24	1.439(3)
C1	C2	1.491(3)	C24	C25	1.175(3)
C1	C22	1.398(3)	C25	C26	1.441(3)
C2	C3	1.379(3)	C26	C27	1.392(3)
C2	C10	1.433(3)	C26	C30	1.392(3)
C3	C4	1.408(3)	C27	C28	1.388(3)
C4	C5	1.363(3)	C28	C31	1.510(3)
C5	C6	1.411(3)	C29	C30	1.389(3)
C6	C7	1.412(3)	C29	C32	1.500(3)
C6	C10	1.426(3)	C33	C34	1.444(4)
C7	C8	1.351(4)	C35	C36	1.478(4)
C8	C9	1.398(4)			

Table S3. Bond Angles for **10**.

Atom	Atom	Atom	Angle/°	Atom	Atom	Atom	Angle/°
C32	O1	C35	114.57(18)	C15	C16	C20	120.3(2)
C31	O2	C33	113.4(2)	C17	C16	C20	117.6(2)
C9	N1	C10	116.9(2)	C18	C17	C16	120.5(2)
C1	N2	C11	120.25(17)	C17	C18	C19	118.1(2)
C19	N3	C20	118.3(2)	N3	C19	C18	124.4(3)
C29	N4	C28	116.26(18)	N3	C20	C12	120.26(19)
N2	C1	C2	114.92(17)	N3	C20	C16	121.1(2)
N2	C1	C22	121.48(18)	C16	C20	C12	118.6(2)
C22	C1	C2	123.42(17)	C11	C21	C23	118.56(19)
C3	C2	C1	117.77(18)	C23	C22	C1	118.60(17)
C3	C2	C10	118.10(19)	C21	C23	C24	120.10(18)
C10	C2	C1	124.08(18)	C22	C23	C21	119.50(18)
C2	C3	C4	122.7(2)	C22	C23	C24	120.39(17)
C5	C4	C3	119.7(2)	C25	C24	C23	178.6(2)
C4	C5	C6	120.1(2)	C24	C25	C26	178.1(2)
C5	C6	C7	121.7(2)	C27	C26	C25	121.9(2)
C5	C6	C10	120.3(2)	C27	C26	C30	117.48(19)
C7	C6	C10	117.9(2)	C30	C26	C25	120.60(19)
C8	C7	C6	119.7(2)	C28	C27	C26	118.9(2)
C7	C8	C9	118.1(2)	N4	C28	C27	124.29(19)
N1	C9	C8	125.6(2)	N4	C28	C31	119.87(19)
N1	C10	C2	119.46(19)	C27	C28	C31	115.77(19)
N1	C10	C6	121.68(19)	N4	C29	C30	123.9(2)
C6	C10	C2	118.84(19)	N4	C29	C32	119.94(18)
N2	C11	C12	114.27(18)	C30	C29	C32	116.13(18)
N2	C11	C21	121.55(18)	C29	C30	C26	119.23(19)
C21	C11	C12	124.13(19)	O2	C31	C28	113.08(19)

C13	C12	C11	117.3(2)	O4	C31	O2	125.2(2)
C13	C12	C20	118.38(19)	O4	C31	C28	121.6(2)
C20	C12	C11	124.3(2)	O1	C32	C29	114.01(18)
C12	C13	C14	122.6(2)	O3	C32	O1	123.7(2)
C15	C14	C13	119.7(2)	O3	C32	C29	122.3(2)
C14	C15	C16	120.3(2)	C34	C33	O2	108.0(2)
C15	C16	C17	122.0(2)	O1	C35	C36	107.6(2)

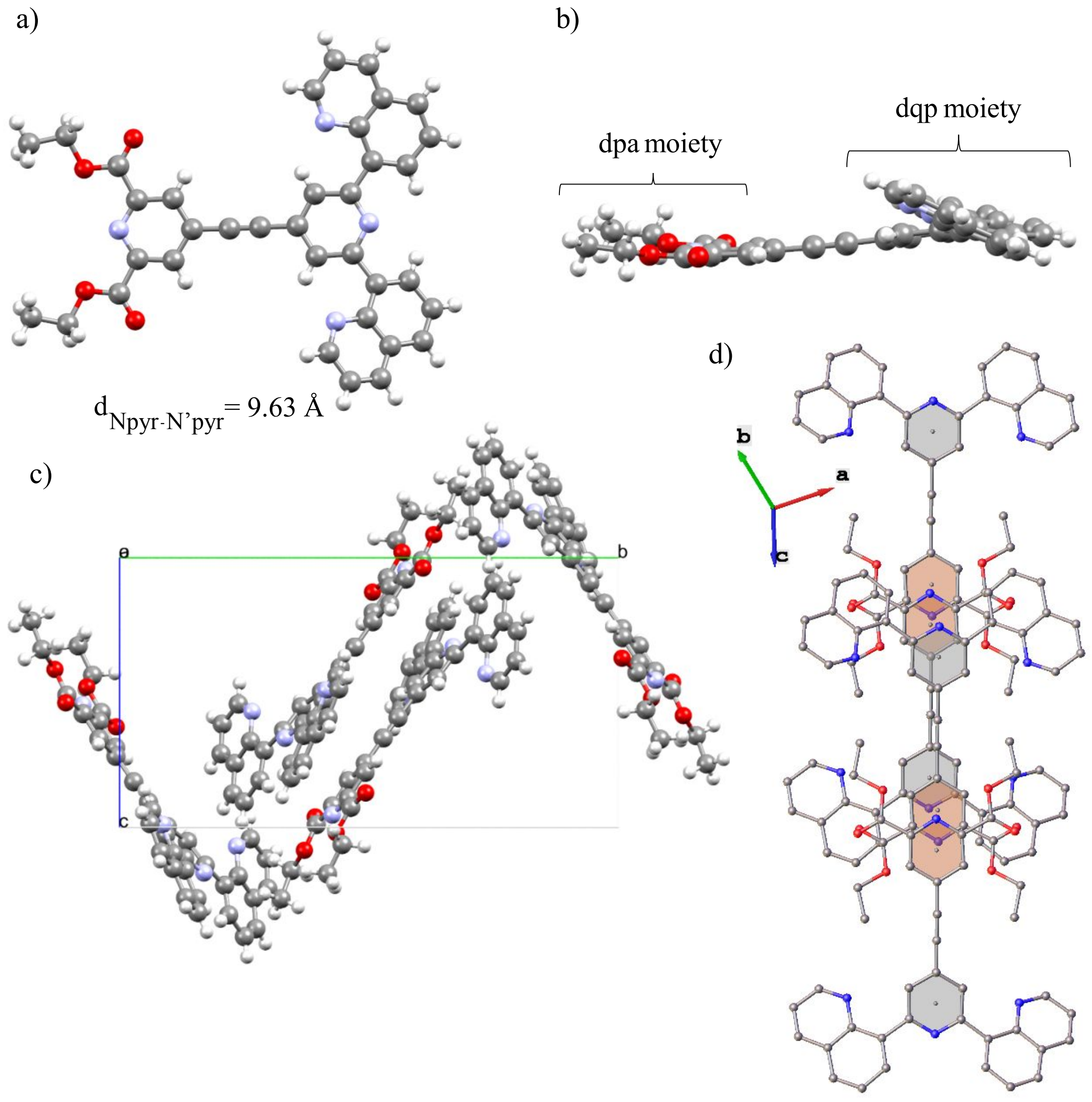

Figure S3. Spatial representation of **10** obtained from crystals suitable for X-Ray Diffraction (XRD). a) Top view of **10**. Colour code: light blue: nitrogen, red: oxygen, grey: carbon, white: hydrogen; b) lateral representation; c) spatial representation of the unit cell. For clarity, only molecules whose centroids fit within the unit cell are represented. d) View of the π- π interactions between the pyridine planes (the diethyl dipicolinate plane is represented in light red and the **dqp** plane in grey).

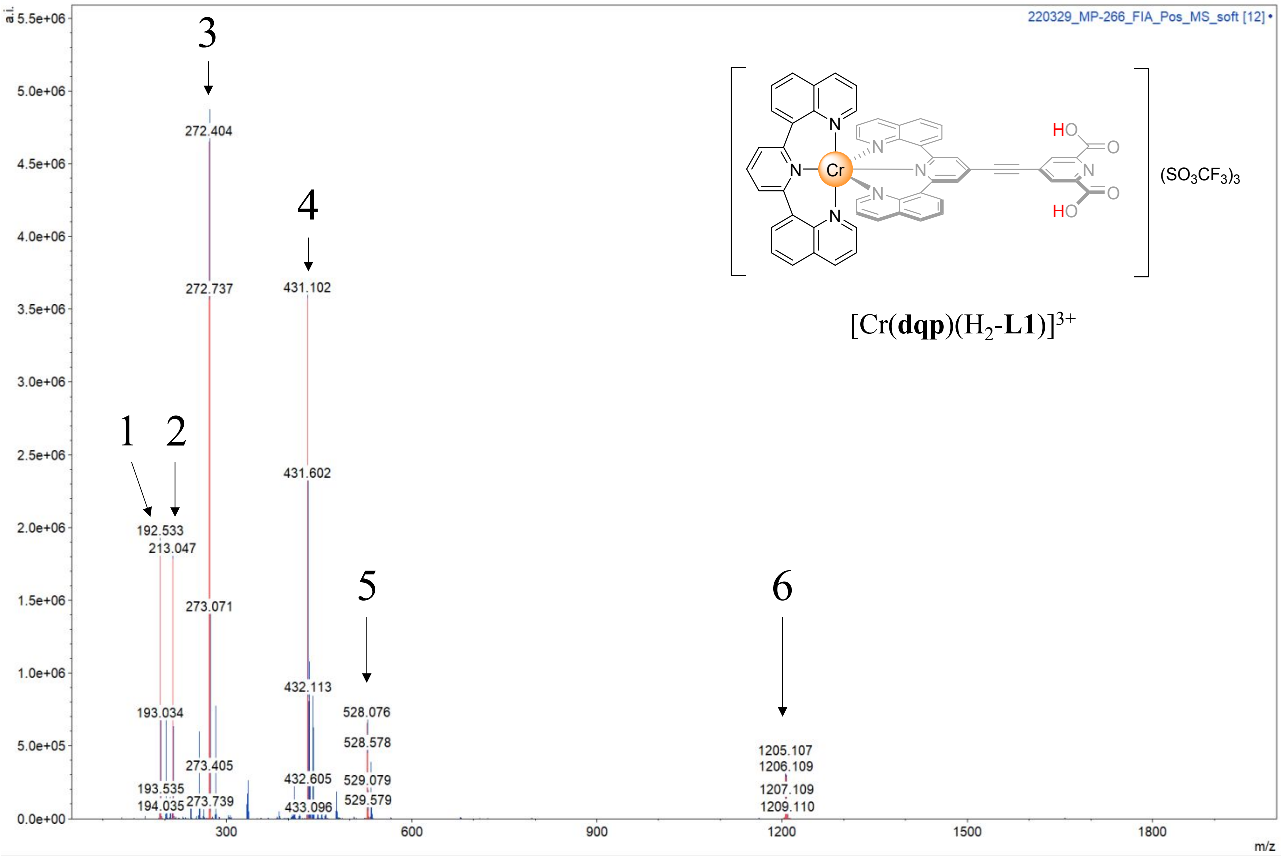

Figure S4. HR-MS ESI-TOF full spectrum of [Cr(**dqp**)(H_2-**L1**)]$^{3+}$. In blue is represented the experimental spectra and in red the theoretical fit.

Table S4. Peak assignment and calculations of selected peak of the HR-MS ESI-TOF spectrum of [Cr(**dqp**)(H_2-**L1**)]$^{3+}$.

Peak	*m/z* experimental	intensity	Relative intensity	*m/z* theoretical	Empirical formula	Assignment
1	192.533	1.46E+06	37.804	192.5330	$C_{23}H_{15}CrN_3^{2+}$	[Cr(**dqp**)]$^{2+}$
2	213.047	1.49E+06	38.759	213.0463	$C_{25}H_{18}CrN_4^{2+}$	[Cr(**dqp**)CH_3CN]$^{2+}$
3	272.403	3.85E+06	100.000	272.4010	$C_{53}H_{31}CrN_7^{3+}$	[Cr(**dqp**)(H_2-**L1**)]$^{3+}$ - 2 (COOH)
	273.070	1.11E+06	28.863	273.0729	$C_{53}H_{33}CrN_7^{3+}$	[Cr(**dqp**)(H_2-**L1**)]$^{3+}$ - 2 (COO)
4	431.101	1.26E+06	32.649	431.1006	$C_{54}H_{32}CrN_7O_2^{2+}$	[Cr(**dqp**)(H_2-**L1**)]$^{2+}$ - (COO)
5	528.075	2.15E+05	5.577	528.0754	$C_{56}H_{33}CrF_3N_7O_7S^{2+}$	[Cr(**dqp**)(H_2-**L1**)]$^{3+}$ + $SO_3CF_3^-$
6	1205.105	1.35E+05	3.495	1205.1036	$C_{57}H_{33}CrF_6N_7O_{10}S_2^+$	[Cr(**dqp**)(H_2-**L1**)]$^{3+}$ + 2$(SO_3CF_3)^-$

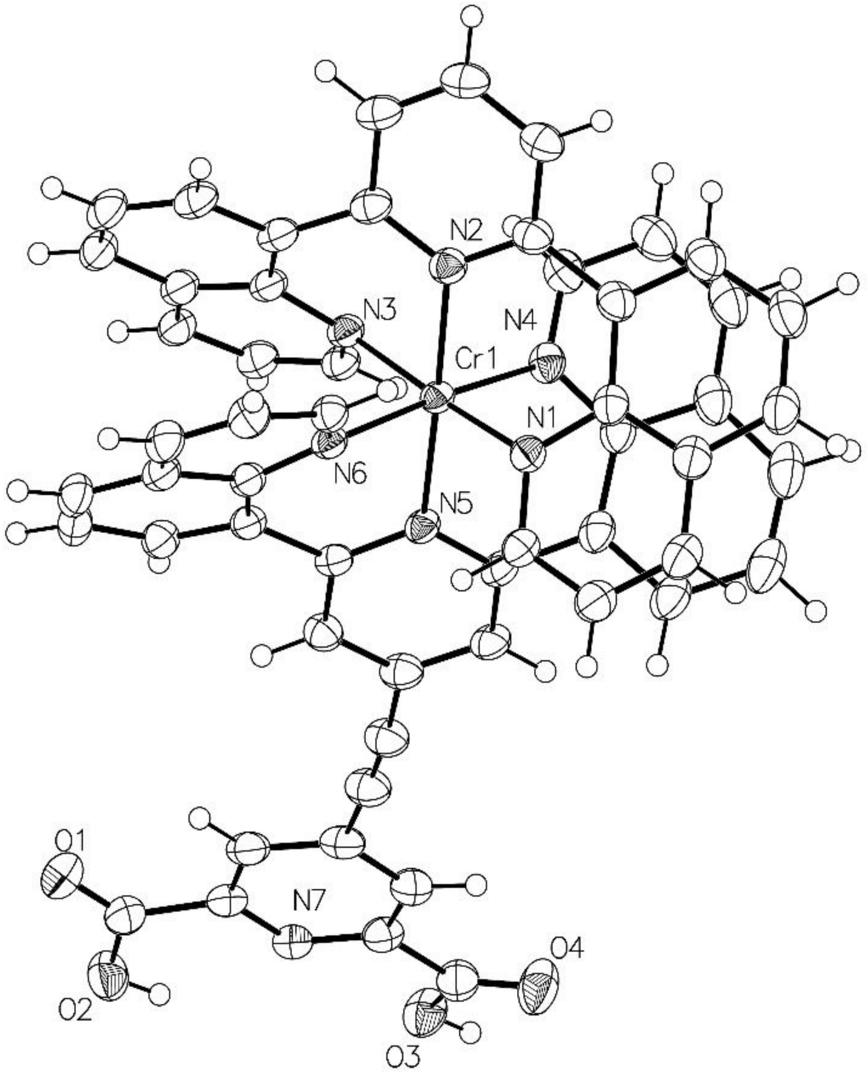

Figure S5. Molecular structure of the chromium complex in the crystal structure of [Cr(**dqp**)(H_2-**L1**)](SO_3CF_3)$_3$. Displacement ellipsoids are shown at 50 percent probability level.

Table S5. Crystal data and structure refinement for [Cr(**dqp**)(H_2-**L1**)](CF_3SO_3)$_3$·3.5H_2O.

**CCDC number**	**2361449**	
Empirical formula	C58 H39.94 Cr F9 N7 O16.47 S3	
Formula weight	1417.52	
Temperature	100.00(16) K	
Wavelength	1.54184 Å	
Crystal system	Triclinic	
Space group	P-1	
Unit cell dimensions	a = 10.8680(2) Å	α= 103.4159(12)°
	b = 13.08986(18) Å	β= 103.2578(15)°
	c = 22.1206(3) Å	γ = 94.1686(15)°
Volume	2952.97(9) Å^3	
Z	2	
Density (calculated)	1.594 Mg/m^3	
Absorption coefficient	3.532 mm^{-1}	
F(000)	1443	
Crystal size	0.136 x 0.079 x 0.053 mm^3	
Theta range for data collection	3.502 to 74.859°.	
Index ranges	-13<=h<=13, -16<=k<=13, -26<=l<=27	
Reflections collected	45863	
Independent reflections	11824 [R(int) = 0.0478]	
Completeness to theta = 67.684°	99.9 %	
Absorption correction	Gaussian	
Max. and min. transmission	1.000 and 0.688	
Refinement method	Full-matrix least-squares on F^2	
Data / restraints / parameters	11824 / 146 / 949	
Goodness-of-fit on F^2	1.028	
Final R indices [I>2sigma(I)]	R1 = 0.0576, wR2 = 0.1601	
R indices (all data)	R1 = 0.0621, wR2 = 0.1632	
Extinction coefficient	n/a	
Largest diff. peak and hole	1.076 and -0.451 e.Å^{-3}	

Comments on the structure model and refinement:

One disordered triflate molecule was refined using two components. The following restraints were used:
SADI S4 C63 S3 C62
RIGU F10 C63 F11 F12 S4 O16 O15 O14
SADI F11 C63 F12 C63 F10 C63 F8 C62 F9 C62 F7 C62
RIGU F7 S3 C62 O13 O12 O11 F9 F8
SADI S4 O16 S4 O14 S4 O15 S3 O12 S3 O11 S3 O13

One water molecule is close to one part of the disordered triflate. Its occupancy was restrained to be the same as the other part of this disordered triflate.
The hydrogen atoms of the COOH functions were visible in the Fourier maps. (A small disorder of the H atom on the O2 position is possible but was not modelled).
The hydrogen atoms of the water molecules containing O20 and O21 O23 were also visible.
For the partly occupied water molecule containing O17 some remaining density was also visible and the hydrogen atoms were put in the position of this density and were allowed to ride on their parent oxygen atom.

A small density is visible still in the density and could be attributed to some partly occupied water molecule. The occupancy refined to about only 10 percent and the molecule was therefore not included in the final model.

Table S6. Selected bond lengths (Å) and angles (°) for [Cr(**dqp**)(H_2-**L1**)](CF_3SO_3)$_3$·3.5H_2O.

**Bond Length/Å**			**Bond Angles/°**			
**Atom**	**Atom**	**Length/Å**	**Atom**	**Atom**	**Atom**	**Angle/°**
Cr1	N3	2.067(2)	N3	Cr1	N4	88.19(8)
Cr1	N6	2.056(2)	N6	Cr1	N3	93.11(8)
Cr1	N1	2.048(2)	N6	Cr1	N4	175.51(8)
Cr1	N5	2.045(2)	N1	Cr1	N3	176.08(8)
Cr1	N4	2.073(2)	N1	Cr1	N6	86.62(8)
Cr1	N2	2.035(2)	N1	Cr1	N4	92.37(8)
			N5	Cr1	N3	89.92(8)
			N5	Cr1	N6	87.57(8)
			N5	Cr1	N1	93.97(8)
			N5	Cr1	N4	88.14(8)
			N2	Cr1	N3	87.58(8)
			N2	Cr1	N6	92.63(8)
			N2	Cr1	N1	88.53(9)
			N2	Cr1	N5	177.50(8)
			N2	Cr1	N4	91.72(9)

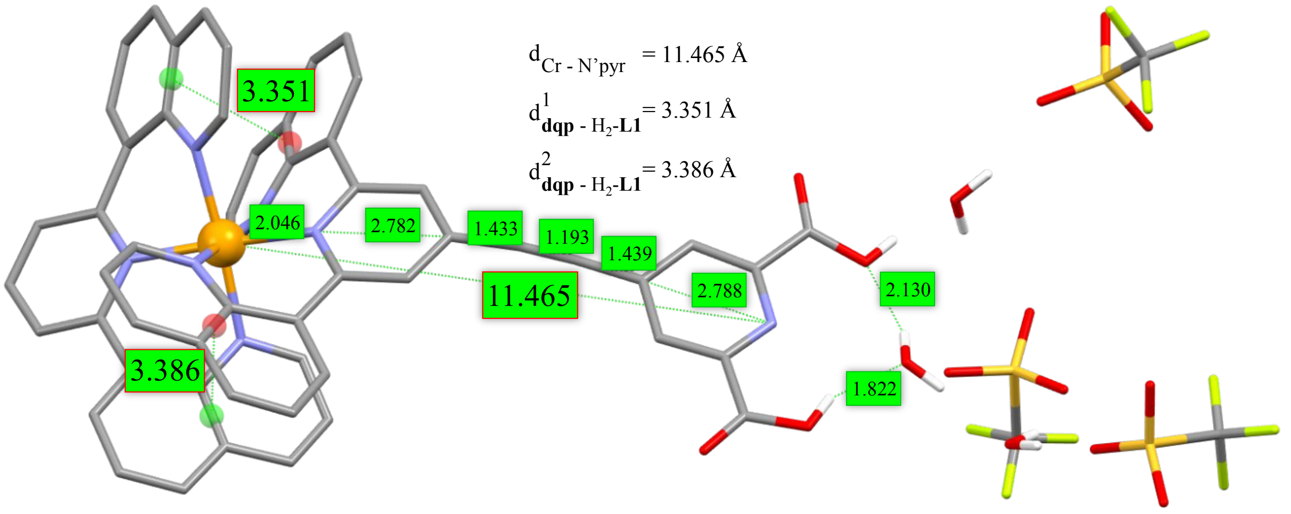

Figure S6. Pertinent distance calculations in [Cr(**dqp**)(H_2-**L1**)](O_3SCF_3)$_3$ in Å. The distances between **dqp** and H_2-**L1**, have been calculated as the distance between each centroid of the 10 atoms of each quinoline (green: dqp centroid; red: H_2-**L1** centroid). For clarity, the hydrogen atoms on the **dqp** moiety are not represented.

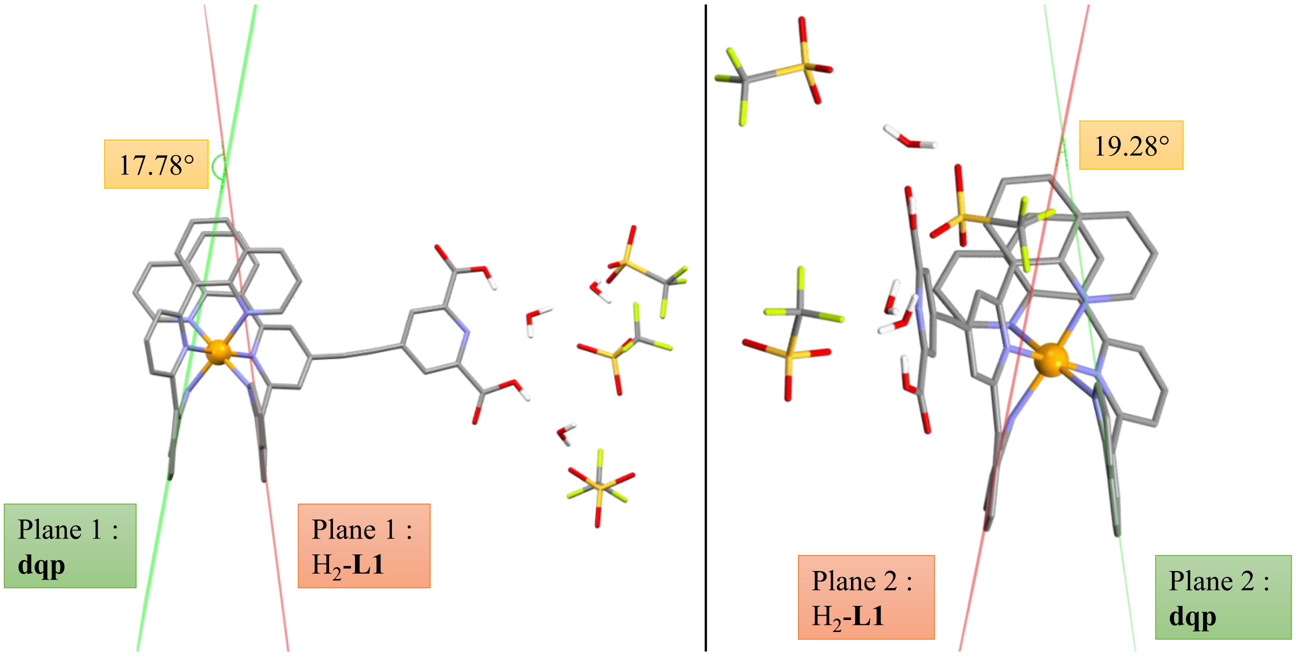

Figure S7. dqp and H_2-**L1** quinoline planes and their respective dihedral angles in [Cr(**dqp**)(H_2-**L1**)]$^{3+}$.

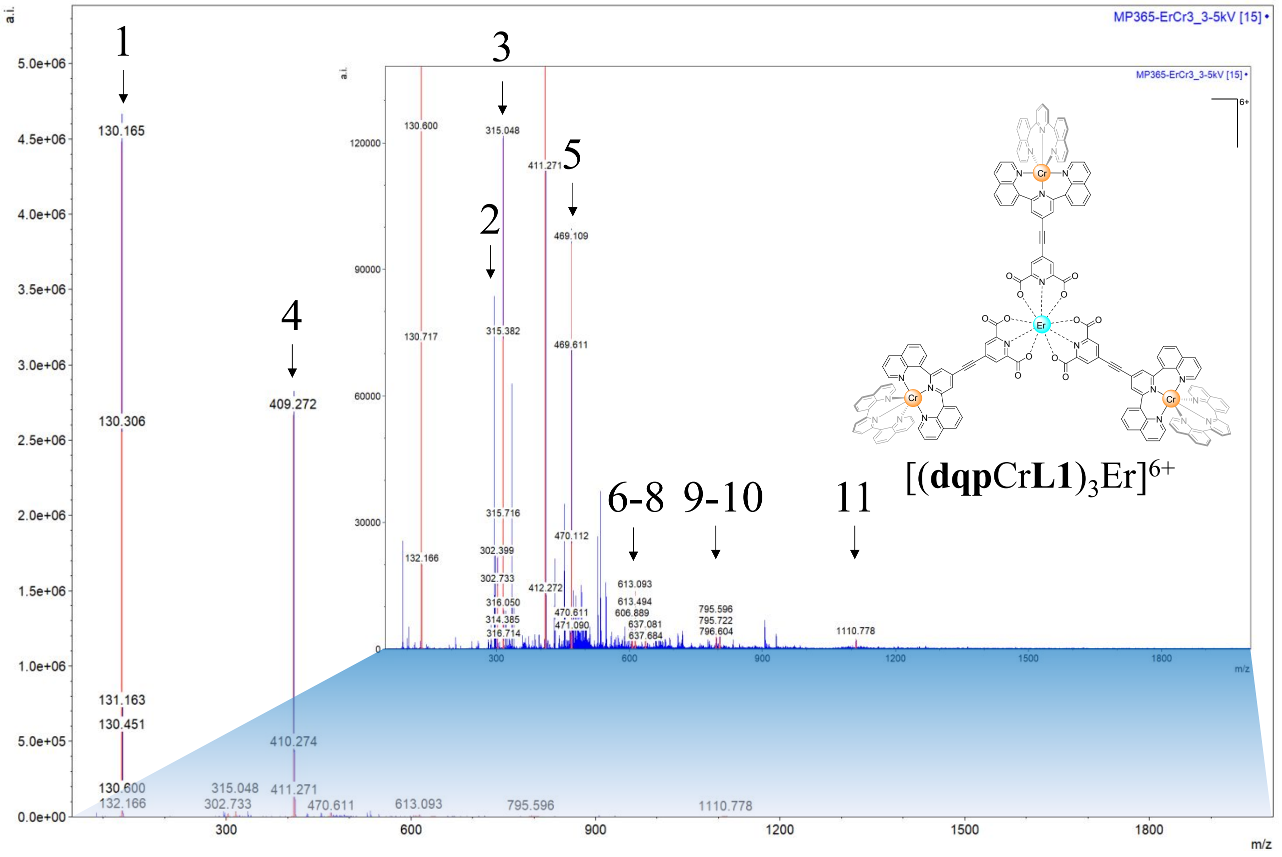

Figure S8. HR-MS ESI-TOF full spectrum of [(**dqp**Cr**L1**)$_3$Er]$^{6+}$ together with a zoom of the full spectrum. In blue is represented the experimental spectra and in red the theoretical fit.

Table S7. Peak assignment and calculations of selected peak of the HR-MS ESI-TOF spectrum of [(**dqp**Cr**L1**)$_3$Er]$^{6+}$.

Peak	*m/z* experimental	intensity	Relative intensity	*m/z* theoretical	Empirical formula	Assignment
1	130.165	4.49E+06	100.00	130.1596	$C_8H_{20}N^+$	H-DIPEA$^+$
				130.1590	$C_{56}H_{133}N_7^{7+}$	7 H-DIPEA$^+$
2	302.399	2.15E+04	0.48	302.3995	$C_{55}H_{33}CrN_7O_4^{3+}$	[Cr(**dqp**)(H$_2$-**L1**)]$^{3+}$
3	315.048	1.21E+05	2.70	315.0515	$C_{55}H_{32}CrKN_7O_4^{3+}$	[Cr(**dqp**)(H-**L1**)]$^{2+}$ + K$^+$
4	409.272	2.67E+06	59.56	409.2712	$C_{17}H_{40}F_3N_2O_3S^+$	2 H-DIPEA$^+$ + $SO_3CF_3^-$
5	469.109	9.61E+04	2.14	469.1086	$C_{56}H_{36}CrN_7O_5^{2+}$	[Cr(**dqp**)(H-**L1**)]$^{2+}$ + MeOH
6	606.889	6.73E+03	0.15	606.6878	$C_{166}H_{93}Cr_3ErF_3N_{21}O_{15}S^{5+}$	[(**dqp**Cr**L1**)$_3$Er]$^{6+}$ + $SO_3CF_3^-$
7	613.093	1.35E+04	0.30	613.0931	$C_{167}H_{97}Cr_3ErF_3N_{21}O_{16}S^{5+}$	[(**dqp**Cr**L1**)$_3$Er]$^{6+}$ + $SO_3CF_3^-$ + MeOH
8	636.678	5.63E+03	0.13	636.6798	$C_{167}H_{94}Cr_3ErF_6N_{21}O_{18}S_2^{5+}$	[(**dqp**Cr**L1**)$_3$Er]$^{6+}$ + 2$(SO_3CF_3)^-$ + H$^+$
9	795.596	7.61E+03	0.17	795.5980	$C_{167}H_{93}Cr_3ErF_6N_{21}O_{18}S_2^{4+}$	[(**dqp**Cr**L1**)$_3$Er]$^{6+}$ + 2$(SO_3CF_3)^-$
				795.7232	$(C_{167}H_{93}Cr_3ErF_6N_{21}O_{18}S_2)_2^{8+}$	2[(**dqp**Cr**L1**)$_3$Er]$^{6+}$ + 4$(SO_3CF_3)^-$
10	803.848	4.03E+03	0.09	803.6046	$C_{168}H_{97}Cr_3ErF_6N_{21}O_{19}S_2^{4+}$	[(**dqp**Cr**L1**)$_3$Er]$^{6+}$ + 2$(SO_3CF_3)^-$ + MeOH
11	1110.447	2.43E+03	0.05	1110.4481	$C_{168}H_{93}Cr_3ErF_9N_{21}O_{21}S_3^{3+}$	[(**dqp**Cr**L1**)$_3$Er]$^{6+}$ + 3$(SO_3CF_3)^-$
				1110.6149	$(C_{168}H_{93}Cr_3ErF_9N_{21}O_{21}S_3)_2^{6+}$	2[(**dqp**Cr**L1**)$_3$Er]$^{6+}$ + 6$(SO_3CF_3)^-$

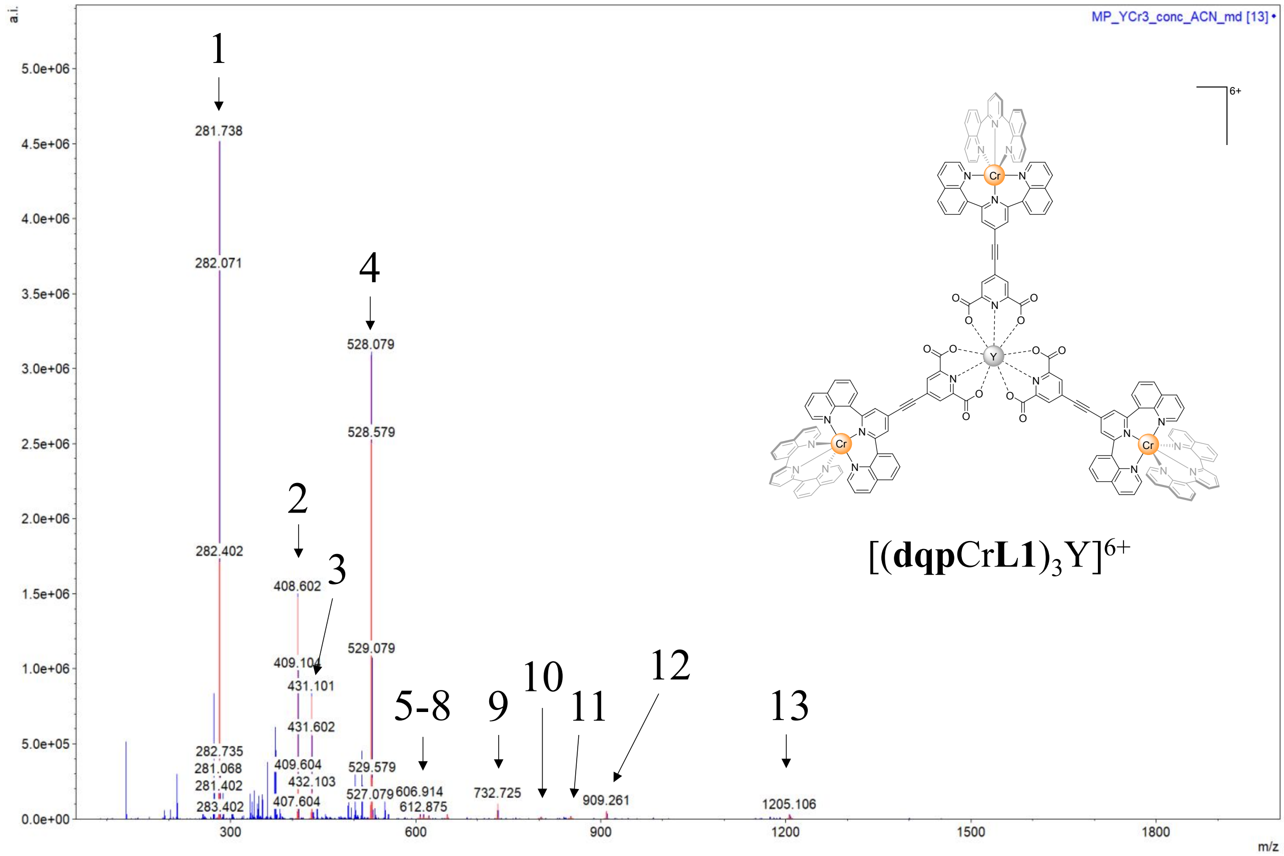

Figure S9. HR-MS ESI-TOF full spectrum of [(**dqp**Cr**L1**)$_3$Y]$^{6+}$. In blue is represented the experimental spectra and in red the theoretical fit.

Table S8. Peak assignment and calculations of selected peak of the HR-MS ESI-TOF spectrum of [(**dqp**Cr**L1**)$_3$Y]$^{6+}$.

Peak	*m*/*z* experimental	intensity	Relative intensity	*m*/*z* theoretical	Empirical formula	Assignment
1	281.738	4.52E+06	100.00	281.7326	$C_{54}H_{31}CrN_7O^{3+}$	[Cr(**dqp**)(H$_2$-**L1**)]$^{3+}$ - COO$^{\cdot}$ - O$^{\cdot}$
2	408.602	1.48E+06	32.83	408.6018	$C_{53}H_{31}CrN_7^{2+}$	[Cr(**dqp**)(H$_2$-**L1**)]$^{2+}$ - 2(COO$^{\cdot}$)
3	431.101	8.17E+05	18.08	431.1006	$C_{54}H_{32}CrN_7O_2^{2+}$	[Cr(**dqp**)(H$_2$-**L1**)]$^{2+}$ - (COO)
4	528.079	3.09E+06	68.43	528.0754	$C_{56}H_{33}CrF_3N_7O_7S^{2+}$	[Cr(**dqp**)(H$_2$-**L1**)]$^{3+}$ + $SO_3CF_3^-$
5	606.914	1.11E+05	2.46	606.9155	$C_{223}H_{124}Cr_4F_9N_{28}O_{25}S_3Y_2^{7+}$	[(**dqp**Cr**L1**)$_4$Y$_2$]$^{10+}$ + 3(SO$_3$CF$_3$)$^-$
6	612.076	3.60E+04	0.80	612.2767	$C_{166}H_{94}Cr_3F_6N_{21}O_{16}S_2Y^{5+}$	[(**dqp**Cr**L1**)$_3$Y]$^{6+}$ - (COO) + 2(SO$_3$CF$_3$)$^-$
7	621.073	2.37E+04	0.53	621.0746	$C_{167}H_{94}Cr_3F_6N_{21}O_{18}S_2Y^{5+}$	[(**dqp**Cr**L1**)$_3$Y]$^{6+}$ + 2(SO$_3$CF$_3$)$^-$ + H$^+$
8	651.066	4.36E+04	0.96	651.0666	$C_{168}H_{95}Cr_3F_9N_{21}O_{21}S_3Y^{5+}$	[(**dqp**Cr**L1**)$_3$Y]$^{6+}$ + 3(SO$_3$CF$_3$)$^-$ + 2H$^+$
9	732.891	9.54E+04	2.11	732.8935	$C_{224}H_{124}Cr_4F_{12}N_{28}O_{28}S_4Y_2^{6+}$	[(**dqp**Cr**L1**)$_4$Y$_2$]$^{10+}$ + 4(SO$_3$CF$_3$)$^-$
10	802.583	1.43E+04	0.32	802.5839	$C_{167}H_{94}Cr_3F_9N_{21}O_{19}S_3Y^{4+}$	[(**dqp**Cr**L1**)$_3$Y]$^{6+}$ - (COO) + 3(SO$_3$CF$_3$)$^-$
11	851.070	1.78E+04	0.39	851.0713	$C_{169}H_{95}Cr_3F_{12}N_{21}O_{24}S_4Y^{4+}$	[(**dqp**Cr**L1**)$_3$Y]$^{6+}$ + 4(SO$_3$CF$_3$)$^-$ + 2H$^+$
12	909.261	5.46E+04	1.21	909.2627	$C_{225}H_{124}Cr_4F_{15}N_{28}O_{31}S_5Y_2^{5+}$	[(**dqp**Cr**L1**)$_4$Y$_2$]$^{10+}$ + 5(SO$_3$CF$_3$)$^-$
13	1205.106	2.88E+04	0.64	1205.1036	$C_{57}H_{33}CrF_6N_7O_{10}S_2^+$	[Cr(**dqp**)(H$_2$-**L1**)]$^{3+}$ + 2(SO$_3$CF$_3$)$^-$

A)

B)

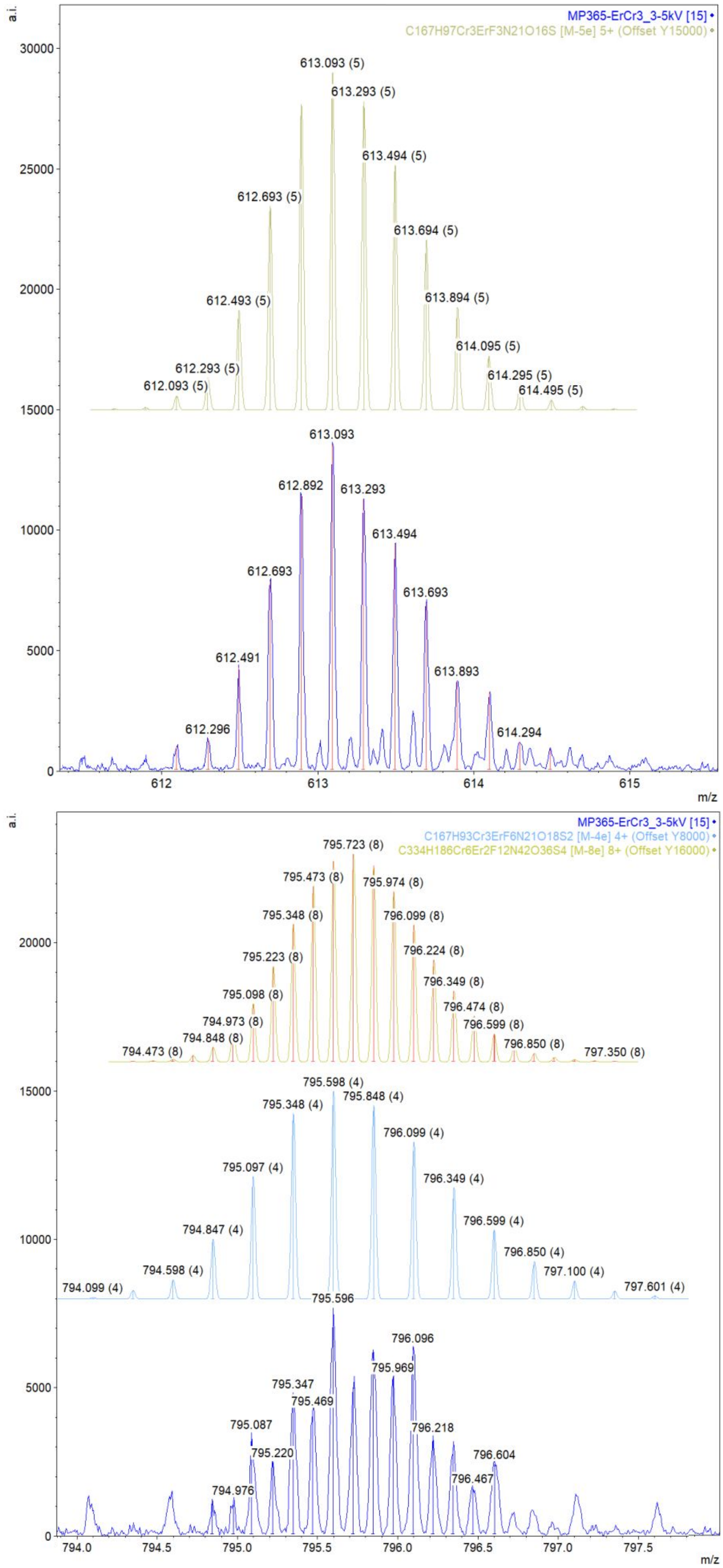

Figure S10. Zoom of peak 7 (A) and peak 9 (B) of [(**dqp**Cr**L1**)$_3$Er]$^{6+}$ with their respective theoretical fit (in green for A and light blue and green for B)

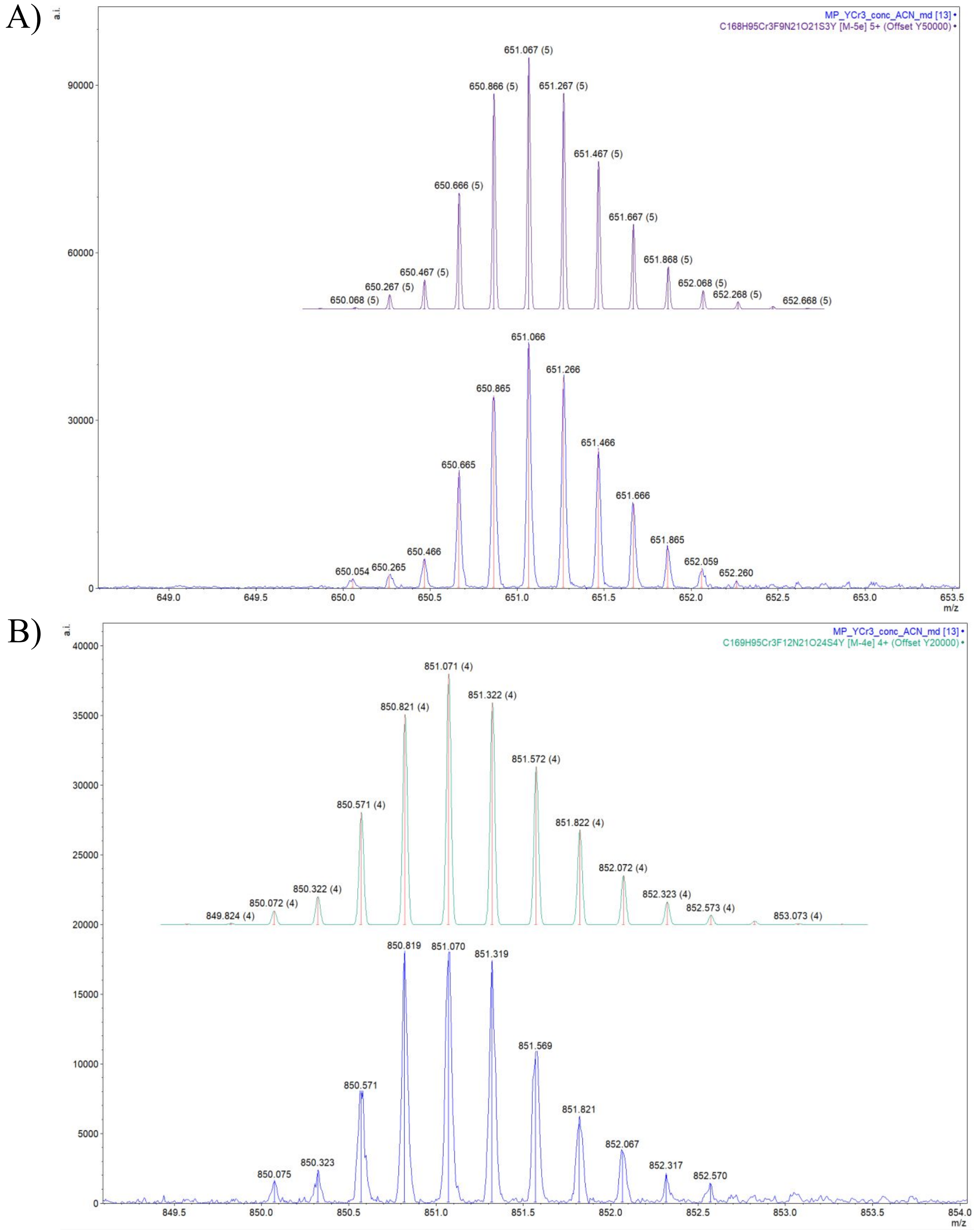

Figure S11. Zoom of peak 8 (A) and peak 11 (B) of $[(\mathbf{dqp}\mathrm{Cr}\mathbf{L1})_3\mathrm{Y}]^{6+}$ with their respective theoretical fit (in purple for A and green for B)

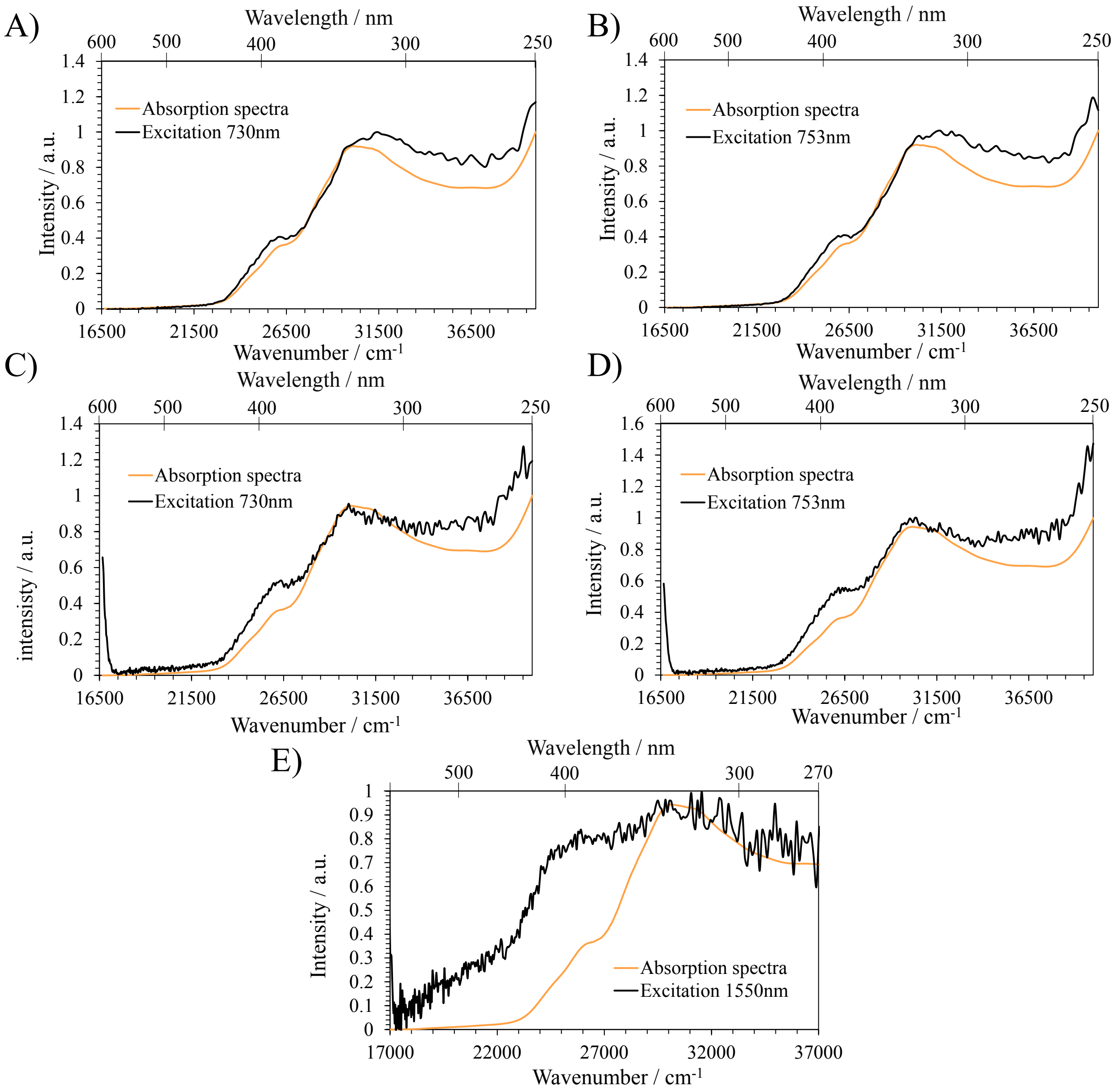

Figure S12. Solid-state excitation spectra recorded for all the emissive transitions in the $[(\mathbf{dqp}\mathrm{Cr}\mathbf{L1})_3\mathrm{Ln}]^{6+}$(Ln = Er, Y) complexes. A) Cr($^2T_1 \rightarrow {}^4A_2$) at 730 nm in $[(\mathbf{dqp}\mathrm{Cr}\mathbf{L1})_3\mathrm{Y}]^{6+}$. B) Cr($^2E \rightarrow {}^4A_2$) at 753 nm in $[(\mathbf{dqp}\mathrm{Cr}\mathbf{L1})_3\mathrm{Y}]^{6+}$. C) Cr($^2T_1 \rightarrow {}^4A_2$) at 730 nm in $[(\mathbf{dqp}\mathrm{Cr}\mathbf{L1})_3\mathrm{Er}]^{6+}$. D) Cr($^2E \rightarrow {}^4A_2$) at 753 nm in $[(\mathbf{dqp}\mathrm{Cr}\mathbf{L1})_3\mathrm{Er}]^{6+}$. E) Er($^4I_{13/2} \rightarrow {}^4I_{15/2}$) at 1550 nm in $[(\mathbf{dqp}\mathrm{Cr}\mathbf{L1})_3\mathrm{Er}]^{6+}$.

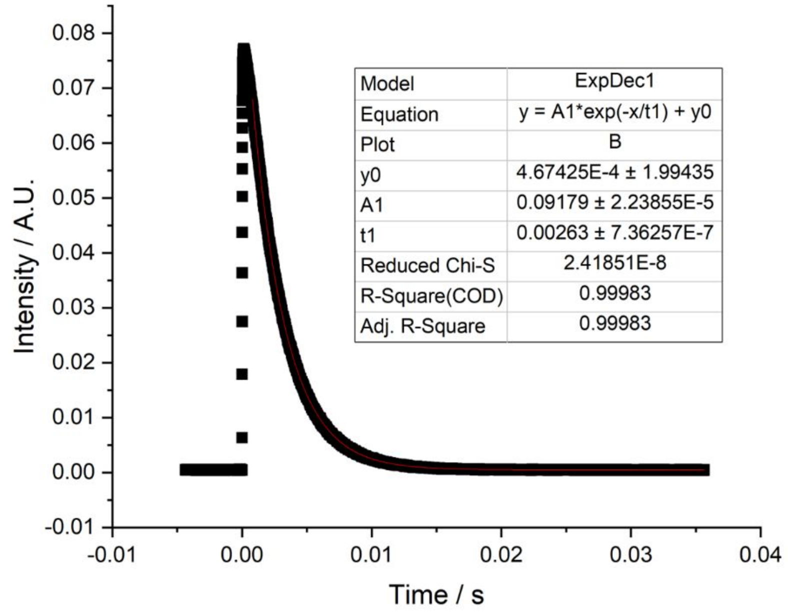

Figure S13. Lifetime measurements of [Cr(**dqp**)(H_2-**L1**)]$^+$ in acetonitrile at room temperature in anaerobic conditions (acetonitrile, 10^{-4} M, 749 nm) using a pulse Nd-YAG laser at 355 nm.

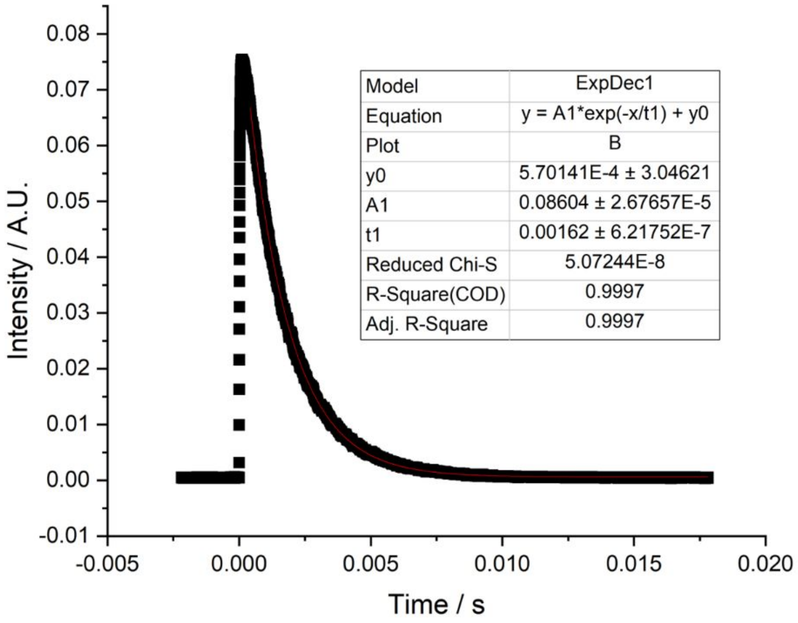

Figure S14. Lifetime measurements of [Cr(**dqp**)(H_2-**L1**)]$^+$ in frozen H_2O/DMSO (1:1) at 77K (10^{-4} M, 749 nm) using a pulse Nd-YAG laser at 355 nm.

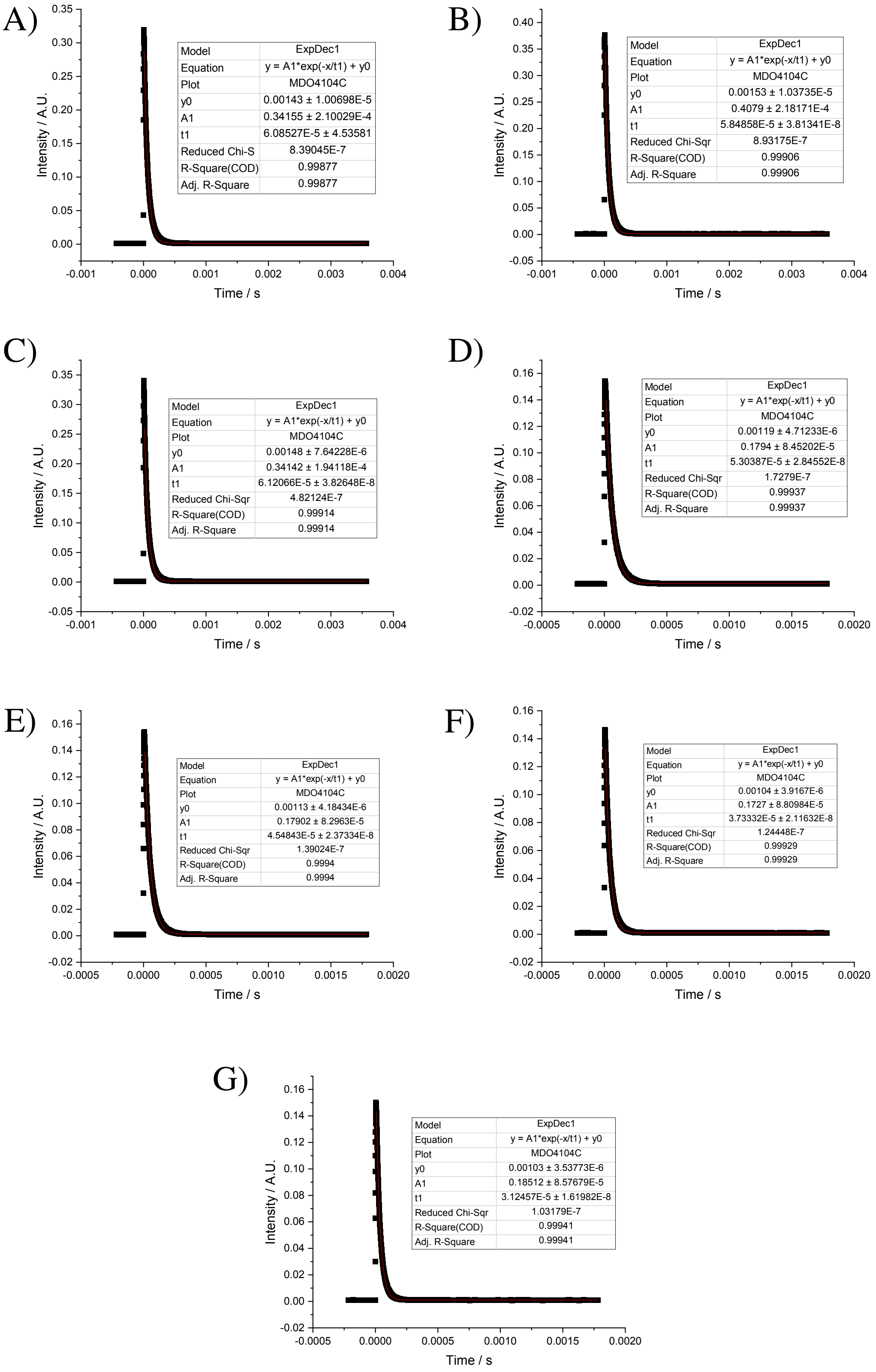

Figure S15. Experimental exponential fitting of the Cr(^{2}E, $^2T_1 \rightarrow {}^4A_2$) excited state lifetime in [(**dqp**Cr**L1**)$_3$Er]$^{6+}$ at 752 nm using a pulse Nd-YAG laser at 355 nm in the solid state. A) 150K; B) 175K; C) 200K; D) 225K; E) 250K; F) 275K; G) 298K.

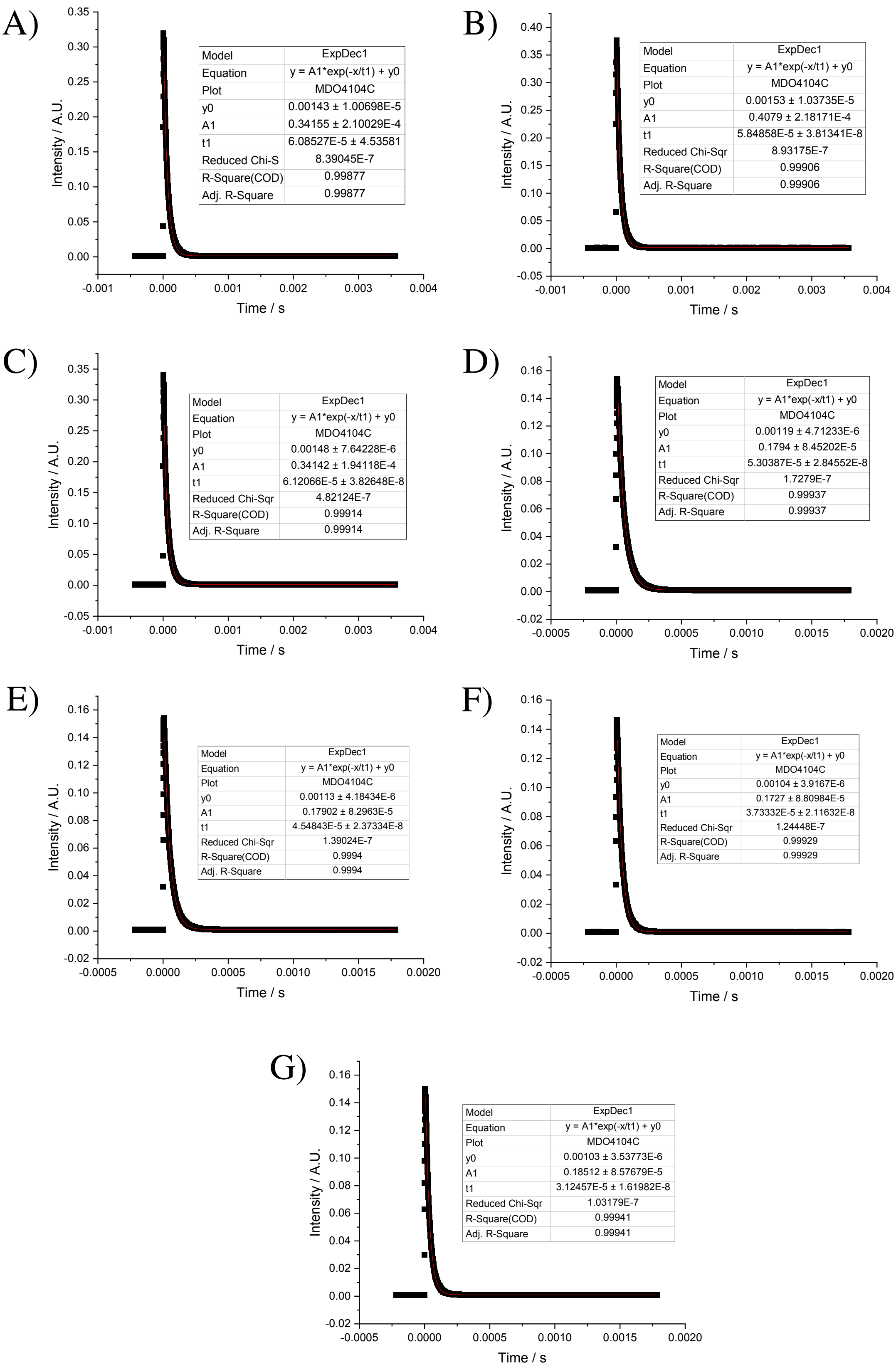

Figure S16. Experimental exponential fitting of the Cr(^{2}E, ^{2}T$_1$ → ^{4}A$_2$) excited state lifetime in [(**dqp**Cr**L1**)$_3$Y]$^{6+}$ at 752 nm using a pulse Nd-YAG laser at 355 nm in the solid state. A) 150K; B) 175K; C) 200K; D) 225K; E) 250K; F) 275K; G) 298K.

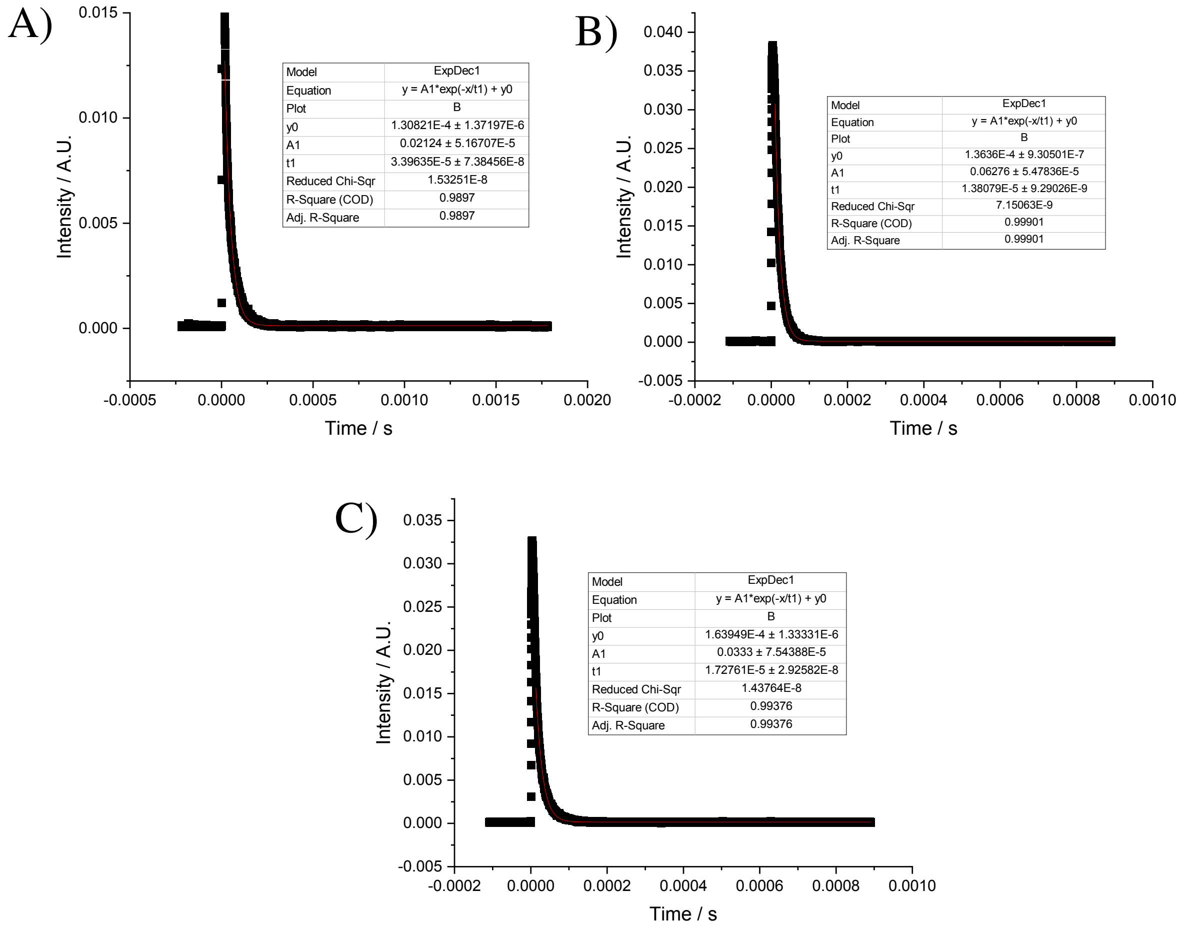

Figure S17. Experimental exponential fitting of the Cr(^{2}E, ^{2}T$_1$ → ^{4}A$_2$) excited state lifetime in [(**dqp**Cr**L1**)$_3$Er]$^{6+}$ at 752 nm using a pulse Nd-YAG laser at 355 nm in acetonitrile at 10^{-3} M. A) 250K; B) 275K; C) 298K.

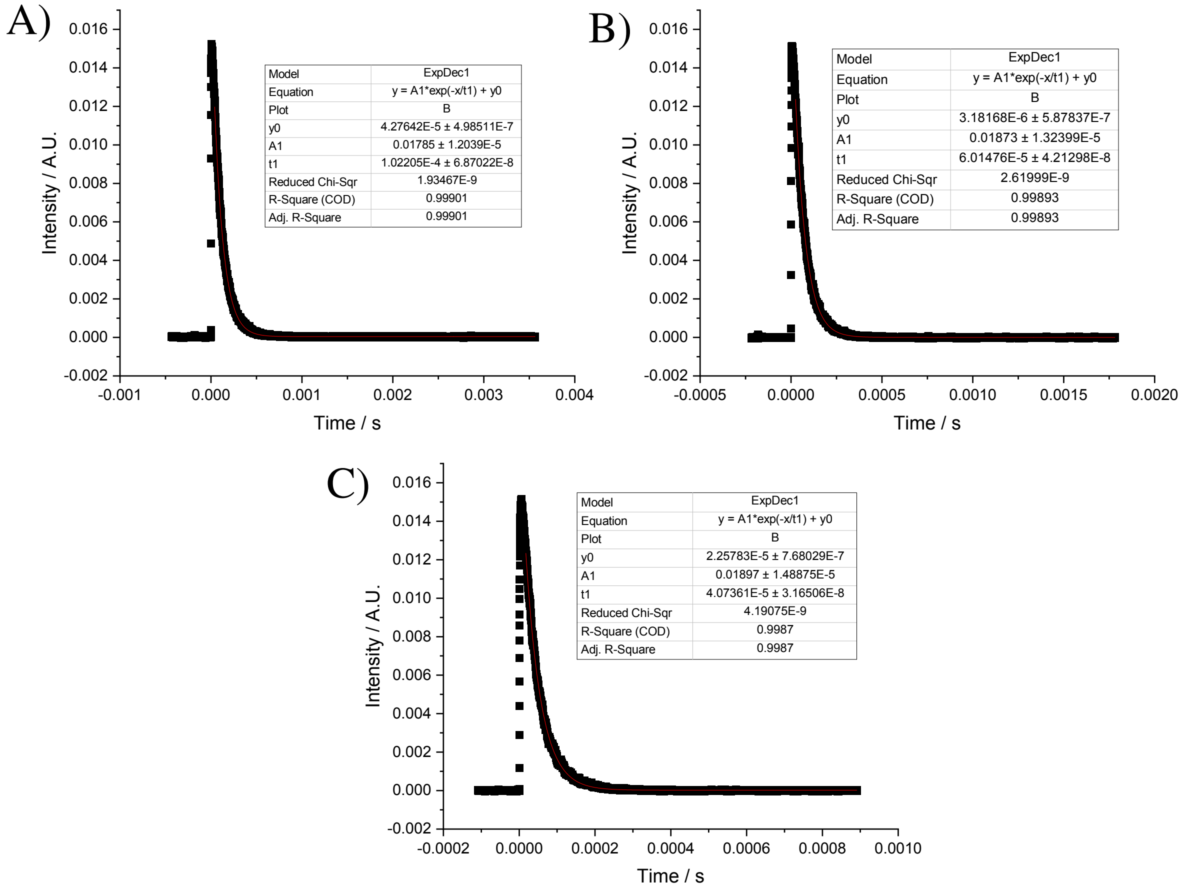

Figure S18. Experimental exponential fitting of the Cr(^{2}E, $^2T_1 \rightarrow {}^4A_2$) excited state lifetime in [(**dqp**Cr**L1**)$_3$Y]$^{6+}$ at 752 nm using a pulse Nd-YAG laser at 355 nm in acetonitrile at 10^{-3} M. A) 250K; B) 275K; C) 298K.

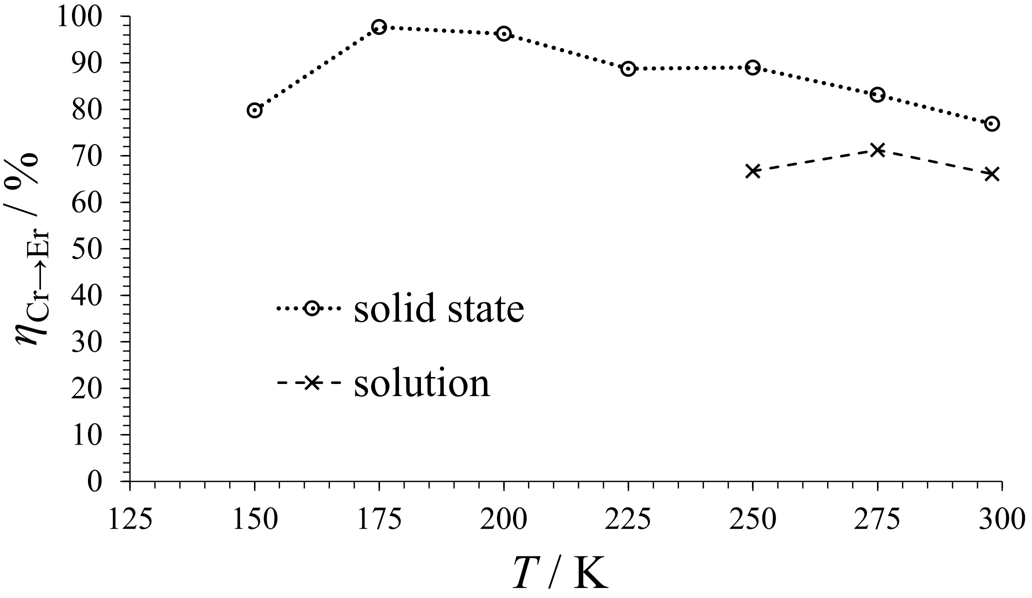

Figure S19. Variation of the EnT efficiency in the [(**dqp**Cr**L1**)$_3$Er]$^{6+}$ assembly as a function of the temperature in solution (liquid range of acetonitrile, 10^{-3} M) and in the solid state.
